# Conversation Analysis of Clients’ Active Resistance to Veterinarians’ Proposals for Long-Term Dietary Change in Companion Animal Practice in Ontario, Canada

**DOI:** 10.3390/ani13132150

**Published:** 2023-06-29

**Authors:** Clare MacMartin, Hannah Wheat, Jason B. Coe

**Affiliations:** 1 Department of Family Relations and Applied Nutrition, University of Guelph, 50 Stone Road East, Guelph, ON N1G 2W1, Canada; 2 Community and Primary Care Research Group, Faculty of Health, University of Plymouth, Plymouth PL4 8AA, Devon, UK; hannah.wheat-1@plymouth.ac.uk; 3 Department of Population Medicine, University of Guelph, 50 Stone Road East, Guelph, ON N1G 2W1, Canada; jcoe@uoguelph.ca

**Keywords:** adherence, communication, compliance, conversation analysis, decision-making, treatment recommendations, nutrition, qualitative analysis, veterinary education

## Abstract

**Simple Summary:**

Research suggests that half of all veterinary clients fail to adhere to veterinarians’ dietary recommendations, which can lead to serious consequences for pet health. However, little is known about what clients’ resistance to such recommendations looks like in actual talk in veterinary consultations and how veterinarians respond. The present study aimed to fill this gap by using conversation analysis to investigate clients’ active resistance to veterinarians’ proposals for long-term changes to cats’ and dogs’ diets in 23 segments from 21 videotaped appointments in Ontario, Canada. Clients responded by suggesting that the proposals themselves or the nutritional modifications were potentially unnecessary, inappropriate, or unfeasible. Justifications were most frequently based on their pets’ food preferences, multi-pet feeding issues, their current use of equivalent health strategies, or their current enactment of the proposed changes. Thus, client resistance occurred when veterinarians did not first gather relevant diet- and patient-related information, solicit clients’ perspectives, or educate them about the benefits of the recommended changes before proposing them. Veterinarians subsequently accommodated clients’ resistance more often when barriers to adherence involved patient- or client-related issues rather than clients’ lack of medical knowledge. Findings provide valuable evidence for developing effective veterinary communication training and practice guidelines in nutritional assessment and shared decision-making.

**Abstract:**

The impact of nutrition on animal health requires effective diet-related treatment recommendations in veterinary medicine. Despite low reported rates of veterinary clients’ adherence with dietary recommendations, little is known about how clients’ resistance to nutritional proposals is managed in the talk of veterinary consultations. This conversation-analytic study investigated clients’ active resistance to veterinarians’ proposals for long-term changes to cats’ and dogs’ diets in 23 segments from 21 videotaped appointments in Ontario, Canada. Clients’ accounts suggested the proposals themselves or nutritional modifications were unnecessary, inappropriate, or unfeasible, most often based on patients’ food preferences, multi-pet feeding issues, current use of equivalent strategies, or current enactment of the proposed changes. Resistance arose when veterinarians constructed proposals without first gathering relevant diet- and patient-related information, soliciting clients’ perspectives, or educating them about the benefits of recommended changes. Veterinarians subsequently accommodated clients’ concerns more often when resistance involved patient- or client-related issues rather than clients’ lack of medical knowledge. The design of subsequent proposals accepted by clients frequently replaced dietary changes in the initial proposals with nutritional or non-nutritional alternatives and oriented to uncertainty about adherence. This study provides evidence-based findings for developing effective communication training and practice guidelines in nutritional assessment and shared decision-making.

## 1. Introduction

In companion animal medicine, the powerful role of nutrition in the prevention and treatment of acute and chronic diseases is well established [1,2,3]. Some gastrointestinal disorders and hepatopathies, along with chronic kidney disease, are diet-sensitive and hence amenable to nutrition-based treatment approaches [4]. The relationship between diet and disease is even more direct in the case of diet-induced health issues; there can be significant health problems involving contaminant ingestion, nutritional deficiencies, and the consumption of excess nutrition [4]. For example, the growing problem of overweight and obesity in cats and dogs [5,6,7,8,9] has been linked to early mortality and health concerns ranging from diabetes mellitus and cardiovascular diseases to osteoarthritis and hepatic lipidosis. [10,11]. It is not surprising, then, that guidelines for assessing patient nutrition in all veterinary appointments with cats and dogs have been published by the American Animal Hospital Association and the World Small Animal Veterinary Association [1,3] and that dietary management of obesity in cats and dogs has assumed a crucial role in their veterinary care [12].

Underutilization and improper utilization of therapeutic diets are longstanding, large-scale problems [13,14]. The American Animal Hospital Association published a major study [13] in 2003, reporting that, in the United States, owners of 9 million cats and 11.6 million dogs with health conditions that could benefit from therapeutic diets failed to provide such diets for their pets or did not do so for the necessary period of time. Ineffective clinician communication about nutritional recommendations in some cases and the failure to make any nutritional recommendations at all in others have been suggested as probable factors [13]. In just over half (55%) of the cases in which veterinarians recommended the use of therapeutic diets for canine and feline patients with associated health conditions, owners bought the recommended food; adherence declined further over time either because owners failed to keep purchasing the therapeutic diet or because they fed their pets additional food, thereby undermining the diet’s impact [13]. Similarly, a survey study conducted in 2004 found that only 12% of cat and dog owners whose pets had been diagnosed with diseases that could be ameliorated through nutritional changes (e.g., cardiac, gastrointestinal tract/hepatic, lower urinary tract, musculoskeletal and periodontal diseases) reported feeding their pets a therapeutic diet [14]. Veterinarians need to learn to communicate more effectively with their clients about their pets’ nutrition [14]; primary health care practitioners play a pivotal part in assessing patient nutrition, educating clients, and supporting client adherence with sound treatment planning and decision-making in the interests of patient well-being [15,16,17]. 

Patient adherence in human medicine has been defined as “the extent to which a patient follows an agreed-on mode of treatment” ([17], p. 51). Studies of doctor–patient communication in human medicine have identified key characteristics of physicians’ communicative activities that are associated with patient adherence [18,19,20,21,22] and positive health outcomes [23,24,25,26]. Adherence with treatment recommendations has been predicted by the quality of physicians’ communication (assessed through patient and observer ratings) and training in communication skills [22]. Positive patient health outcomes have been linked with highly informative communications by physicians, the clarity of their recommendations, and a high degree of doctor–patient collaboration in treatment decision-making [24].

Although much less communication research on veterinarian–client treatment decision-making has been conducted in veterinary medicine, findings point to implications similar to those in human medicine [13,27,28,29]. Ineffective veterinary communication can limit client understanding of the health benefits of practitioners’ treatment recommendations and can reduce clients’ belief in their importance for patients [13,27,28]. Higher client adherence with medication-related treatment has been associated with the amount of time veterinarians spend with clients [28,29], written instructions and demonstrations regarding medication use [29], and follow-up calls and reminders [29].

A limitation of such research is its reliance on retrospective reporting by clients to identify the important characteristics of treatment-related communication that serve to support subsequent adherence. One study avoided this problem by studying recordings of actual veterinary communication [30]. Variables from the Roter interaction system (RIAS) adapted for the study of veterinarian–client–patient interaction were used to code videotaped interactions involving surgery and dentistry recommendations during companion animal consultations to determine which variables might predict subsequent adherence (measured by information kept in clinic medical files) and client satisfaction (captured in a post-consultation questionnaire) [30]. While overall client adherence was only 30%, it was associated with longer visits, more frequent positive statements by veterinarians toward clients, more sympathetic/empathetic and non-rushed/non-hurried communication by veterinarians, higher proportions of client-centred talk, and higher client satisfaction ratings [30]. Another factor strongly predictive of client adherence in the study was the clarity of veterinarians’ treatment recommendations (as assessed by the researchers); clear recommendations for surgical and dental procedures were seven times more likely to result in client adherence than were ambiguous recommendations [30]. 

The RIAS study [30] is laudatory in its investigation of actual talk rather than relying on informants’ retrospective reporting of prior treatment discussions. However, the generation of composite variables in the RIAS framework necessarily removed information about the possible impact of sequential context on treatment decisions. An inspection of the details that might have been implicated in later adherence (e.g., how the recommendations were specifically designed, how clients responded, and how veterinarians took up clients’ responses) was not possible using this method. Hence, little is known about how treatment discussions, including those related to patient nutrition, unfold turn-by-turn as veterinarians and clients interact with each other.

But such features may be crucial. The 2009 follow-up study [28] to the 2003 American Animal Hospital compliance study [13] suggested that there had been a fundamental misunderstanding among veterinary healthcare providers in the earlier study; “doctors and staff alike confused telling clients what to do with having the client *accept* the recommendation and follow through on it” ([28], p. 16, emphasis added). Furthermore, in her discussion of adherence in veterinary medicine, Abood [17] emphasized that the mode of treatment followed must be something previously agreed upon by both the client and clinician. But how is veterinarian–client agreement or disagreement co-constructed in actual treatment discussions, including those about dietary modification? What do verbal displays of client resistance to veterinarians’ nutritional recommendations look like and on what grounds do clients resist such recommendations? How do veterinarians respond to client resistance, and how do these responses and ensuing interactions between veterinarians and clients shape the ultimate trajectory of diet discussions and resulting treatment plans? Knowledge about real-life verbal interactions between veterinarians and clients, including nutrition discussions and their outcomes, can be a tool in the development of evidence-based, effective communication curricula in veterinary medical education and sound guidelines for clinical practice by current professionals. 

Conversation analysis is a form of qualitative research that can answer the above questions. It has long been employed to investigate healthcare practitioner–patient interactions in human medicine [31], including treatment recommendations [32,33,34,35,36,37,38,39,40,41,42] and healthcare advice-giving [43,44,45,46]. For example, conversation analysis of physician–patient interactions has demonstrated a pattern of maximizing agreement and minimizing disagreement between physicians and patients in the negotiation of treatment recommendations; a patient withholding a response for two seconds after the physician’s treatment proposal or a patient’s weak acceptance can result in the physician immediately revising the nature of the proposal in subsequent turns to elicit patient agreement [31,47]. Nonadherence in human medicine has been studied by conversation analysts who examine the interactional phenomenon of active resistance where patients question, challenge, or otherwise reject medical recommendations [36,38,39,41,42]. Because clinicians and patients have been shown to orient closely to each other’s contributions in ways that shape the emerging treatment discussion, treatment recommendations are not viewed by conversation analysts as actions attributable to physicians alone at one point in a sequence of talk but rather as joint social action, involving both physician and patient, unfolding over time in multiple turns [36,40,47]. Increasingly, conversation analysis has been applied to the study of veterinary communication in companion animal practice [48,49,50,51,52], most recently in the areas of dietary history-taking [51] and nutritional recommendations [52]. 

The present study is part of a larger project that employed conversation analysis to study veterinarian–client interactions in the negotiation of veterinarian-initiated long-term changes to cats’ and dogs’ diets in companion animal clinics in Ontario, Canada [52]. We adopted a broad definition of the phenomenon under study in that we investigated not only instances of clear advice on a single preferred course of action recommended by the clinician, but also proposals that discussed multiple options. In a previous publication [52], we reported on the linguistic design of the nutritional proposals initiated by veterinarians, the types of changes proposed, and the health concerns targeted, along with the phases of the visits during which the proposals were introduced. 

Very few proposals (4%) took the conventional form of “I would recommend…”; the most frequent designs (25%) involved descriptions of options, actions, or foods (e.g., “There are also special diets….”) [52]. The prescriptiveness of the proposals was often reduced through various grammatical and lexical features such as the use of modals (e.g., “may”; “might”), qualifying adverbs (e.g., “maybe”), and verbs that were oriented to attempting a food change (e.g., “try”) or to merely considering one (e.g., “One thing you may wanna consider…”) [52]. Thus, veterinarians mitigated their own authority to impose nutritional changes on clients and patients; by reducing the certainty with which adherence with proposed changes could be presumed, veterinarians’ proposals implicitly or explicitly oriented to various contingencies that could affect adherence [52]. Veterinarians mentioned multiple product options for clients when proposing nutritional changes requiring the purchase of new food, rather than promoting veterinarian-only therapeutic diets exclusively [52]. 

The present study builds on those findings by reporting on one strand of our analyses of clients’ responses to veterinarians’ initial proposals for longer-term dietary change: clients’ displays of active resistance to veterinarians’ proposals. Because of the potential health consequences for patients of nonadherence to veterinarians’ dietary proposals, we analyzed the sequential organization and linguistic design of such responses. Specifically, we investigated how clients disaffiliated with (i.e., resisted) veterinarians’ proposals, the grounds on which clients justified their potential nonadherence with proposed nutritional modifications, and what happened subsequently. That is, we also investigated veterinarians’ responses to client resistance and the unfolding trajectories and outcomes of the resulting veterinarian–client discussions. 

## 2. Materials and Methods

### 2.1. Data Archive

**Figure 1:**
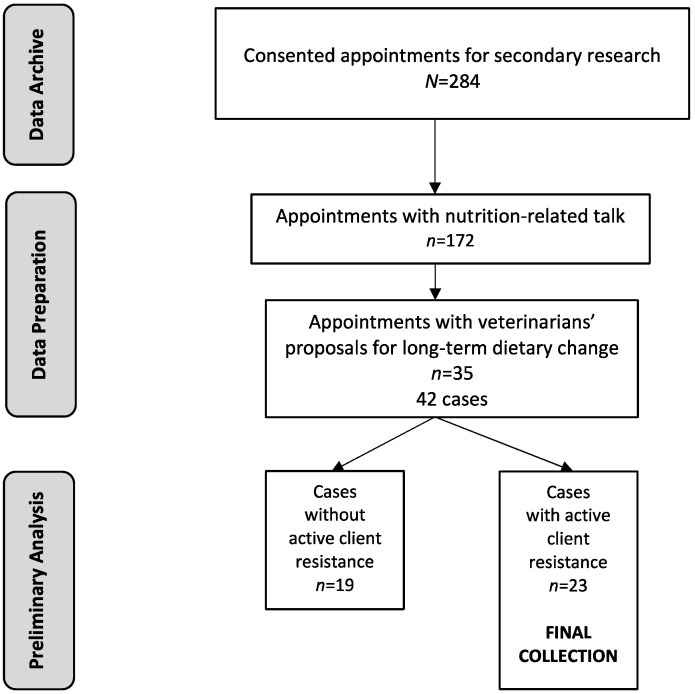
Data preparation, preliminary analyses and development of final collection of cases of active client resistance to veterinarians’ proposals for long-term dietary change.

The data used in the present study were drawn from a set of 350 videotaped appointments collected in 2006 involving 20 veterinarians and their clients in clinics in 14 counties in Ontario, Canada for quantitative research on veterinarian–client–patient communication [53]. Details about the design of the original study have been previously provided [53]. Seventeen of the 20 clinicians and their clients consented to the archiving and use of their videotapes (284 consultations in total) for subsequent secondary research, approved by the University of Guelph Research Ethics Board (see Figure 1).

### 2.2. Data Preparation

Creation of the collection of segments containing clients’ active resistance to veterinarians’ proposals for long-term nutritional change began with a screening of the 284 consented visits available for secondary data analysis (Figure 1) to flag only those in which veterinarians and clients talked about patient diet. We identified 172 such appointments (Figure 1). These appointments comprised 61% of the consented archive available for analysis. Basic orthographic (i.e., word-for-word) transcription was performed on all diet-related talk. Pseudonyms for the proper names of geographic locations, people, and pets were used to anonymize the data. 

Long-term nutritional modifications to patients’ diets were discussed in 55 (32%) of the 172 visits containing nutritional talk (Figure 1). Long-term dietary changes were selected as the focus of analysis of client resistance because of the significant impact long-term nutritional modifications could have on patient health. Proposed changes variously involved the addition or replacement of current main foods or treats and/or the cessation of current favoured foods and treats (including cow’s milk), details of which have been provided elsewhere [52].

Proposals initiated by veterinarians rather than clients were chosen for conversation analysis because of the possible challenges for clinicians in proposing such alterations and the need to initiate such recommendations to support patient health [52]. Among the 55 appointments containing long-term dietary proposals, 35 (64%) involved veterinarians initiating the proposals rather than clients (Figure 1). Transcripts of dietary discussions in the 35 appointments were used to identify 42 discrete segments in which veterinarians proposed to clients some type of long-term alteration to patients’ diets (Figure 1). There were more segments than appointments because some appointments contained multiple kinds of nutritional proposals. The 42 segments included a broad spectrum of proposals. These ranged from recognizable recommendations or suggestions to adopt a singular course of action calling for client acceptance or rejection (e.g., “So she should be getting onto…”; “I would recommend definitely trying to…”; “we could put him on…”) to descriptions of diets or nutritional options possibly hearable by clients as merely information-sharing [52].

### 2.3. Characteristics of Appointments Containing Veterinarian-Proposed Long-Term Dietary Change

In the collection of 35 appointments in which veterinarians proposed long-term nutritional modifications, 15 (88%) of the original 17 practitioners in the consented archive were retained (10 women and five men). The median length of time in veterinary practice was 10 years (range, 2 to 25); 14 of the veterinarians practiced in clinics in which two or more veterinarians worked. Of the 15 clinics included in the present study, seven were in urban areas, five in suburban areas, and three in rural areas. In 18 (51%) of the 35 appointments, patients were dogs and in 17 (49%) appointments, patients were cats. Regarding the type of appointment, 25 (71%) of the 35 were wellness visits, nine (26%) were problem visits, and one (3%) was a follow-up visit. Wellness visits were regular appointments scheduled for routine and preventative care of apparently healthy patients, while problem visits involved special appointments scheduled to address injury or illness in sick patients. 

### 2.4. Analytic Method: Conversation Analysis

Conversation analysis involves the qualitative study of audio and video recordings of naturally occurring conversations along with extremely detailed, specially notated transcripts of those recordings to identify the social actions that are being performed [54].

Language and social interaction have been shown to be deeply ordered in nature; the study of patterns of interaction between speakers across a sequence of turns taken by different speakers during a conversation is the typical focus of conversation-analytic research [55,56,57,58], including the present study.

In line with the methodological procedures of conversation analysis, for a past study [52] and the present one, we repeatedly listened to and observed the videotaped dietary proposal segments [55,56,57,58], further refining the word-for-word transcripts using special transcription notation [59,60] that captured features like overlaps during which more than one person was speaking, gaps between stretches of talk, shifts in speech volume and speed, and changes in vocal intonation. Additional information about nonverbal activities (e.g., head nods, eye gaze) was also incorporated on the transcripts as potentially consequential for the investigation of client resistance.

Conversation analysis has identified a basic sequence in interaction called an *adjacency pair* [60] whereby a first turn taken by one speaker (the *first pair part*) expects a particular sort of responsive action in the form of a second turn by another speaker (the *second pair part*) [61]. An easily recognizable type of adjacency pair involves a question–answer sequence whereby, for example, a veterinarian’s question to a client about the contents of a pet’s diet expects an answer to that question [51]. If an answer is not forthcoming, the smooth progression (or *progressivity*) of the conversation is threatened [61]. Similarly, a medical treatment recommendation is the first pair part in an adjacency pair sequence calling for the patient to accept or reject (the second pair part) the recommendation; if a patient fails to accept or reject the recommendation, the physician may modify the recommendation, provide justifications for it, and/or otherwise pursue acceptance from the patient [31]. 

A crucial concept in the analysis of adjacency pair sequences in the present study involves *preference* [62], whereby a recipient of a recommendation or proposal tends to minimize overt rejection of the proposal as demonstrated in the sequential structure and the design of their response [61,62]. It is important to note that “preference” refers not to individual internal preferences, but rather to principles that are culturally shared and observable in action by examining orderly patterns of talk in line with those principles [62]. Thus, although a recipient of a proposal can either accept or reject it, the two types of responses are non-equivalent; the preferred response to a proposal is acceptance and the dispreferred response is rejection [61]. Preferred responses tend to be delivered more rapidly than dispreferred responses and are typically shorter and less elaborated in design than are dispreferred responses, which are often delayed and accompanied by accounts [61].

Two additional important conversation-analytic concepts informing the present study are the domains of *epistemics* [63] and *deontics* [64]. “Epistemics” refers to the “distribution of rights and responsibilities regarding what participants can accountably know, how they know it, [and] whether they have rights to describe it” ([63], p. 16). “Deontics” refers to “the right to determine others’ future actions” ([64], p. 297). Parallel to descriptions of human medical encounters [42], veterinarians’ epistemic primacy is grounded in their expert medical knowledge, including the ability to interpret clinical signs in their patients, to diagnose, and to know what tests and treatments could be helpful; their deontic authority is based on their rights to make treatment recommendations, such as dietary modifications, and to prescribe and dispense drugs, the latter involving legal control. Clients’ epistemic primacy is based on knowledge of their own preferences, of patients’ preferences and experiences, including patients’ illnesses, and on their own perceptions of medical aspects such as diagnoses and nutrition. Similar to the agency that parents exercise regarding their children as patients in pediatric medicine [42], veterinary clients’ deontic authority is grounded in their right to make healthcare decisions on behalf of animal patients, including whether to agree to certain tests, to fill out and administer prescription drugs, and to purchase food items and feed patients. The deontic authority of clients’ access to nutritional resources is thus somewhat different than that involving access to prescription medications, which are regulated and can legally be obtained only from veterinarians. 

It is important to note that epistemic primacy and deontic authority are not exercised in a static manner; conversation analysts differentiate between *status*, which refers to the enduring elements of knowledge and agency participants possess, and *stance*, by which a participant’s expressions of knowledge and agency in conversation can vary from moment to moment [65,66]. For example, although a veterinarian demonstrates their deontic authority in announcing a treatment recommendation, such as switching to a therapeutic diet, the way they design such a recommendation for a client may invoke a stance that downgrades their own authority to impose such a change (e.g., “You may wanna consider…” [52]. 

We studied both the position [61,67] and composition [68] of veterinarians’ and clients’ turns at talk in those segments in which veterinarians proposed long-term changes to patients’ diets. Drawing on previous conversation-analytic work on the negotiation of treatment proposals in human medicine [32,33,34,35,36,37,38,39,40,41,42,43,44,45,46,47], including research on patient resistance [34,35,36,38,39,41,42], we analyzed clients’ responses to veterinarians’ proposals in terms of the timing, grammatical design, and wording of clients’ responses with a particular focus on cases of clients’ active resistance towards veterinarians’ dietary proposals and how veterinarians responded to client resistance. In order to determine the final outcomes of dietary decision-making, we also reviewed subsequent portions of the relevant videotaped visits and transcripts for any further veterinarian–client interactions relevant to the dietary proposals and/or alternative non-nutritional strategies.

### 2.5. Identification and Categorization of Clients’ Responses

To finalize the collection of active resistance cases as the phenomenon of interest for the present study, we first needed to exhaustively identify and categorize all types of clients’ responses to veterinarians’ proposals. Within the 42 segments involving veterinarian-initiated proposals for long-term dietary change, varying types of client responses following the proposals were identified. Sometimes, more than one type of response occurred in the same proposal segment. Clients were variously found to accept the proposals; withhold acceptance of them in a display of passive resistance by offering acknowledgment tokens, head nods, or continuers like “Mm hm” that enact a listener stance [36,43]; remain silent/fail to take up a proposal with a nutrition-related response; and/or demonstrate active resistance to the proposals, sometimes after initially accepting or withholding acceptance of a previously uttered proposal (see [35,41]).

In order to arrive at a final collection of active resistance cases, once the various types of client responses had been identified, we dichotomized segments and appointments in terms of whether they did or did not contain active resistance by clients (Figure 1). Drawing on definitions of patients’ active resistance in conversation-analytic research in human medicine, e.g., [35,36,41], active resistance in the present study was defined as lexical content introduced by a client in the form of a question or comment that implicitly or explicitly provided an account for the possible non-acceptance of the proposal. We also investigated the characteristics of those appointments containing active client resistance and compared them with the characteristics of those appointments containing no active resistance. The aim was to gather as much contextually relevant information as possible because of the important implications of active resistance for the phenomenon of nonadherence. Similarly, so as to situate active resistance in the broader context of the range of possible client responses to a veterinarian’s proposal, a brief description is provided below of the preliminary analyses of clients’ responses in those segments lacking active resistance. 

#### 2.5.1. Cases without Active Client Resistance 

In the total set of 42 proposal segments, 19 (45%) of cases contained no instances of active resistance (Figure 1). Response types included acceptance of the proposal, passive resistance/withholding of acceptance, no response, and one ambiguous response. For instances to be classifiable as client acceptance cases, the proposals needed to have been unequivocally designed as veterinarians’ recommendations or suggestions calling for client acceptance or declination (e.g., “And she should be on a large breed puppy food”). 

In 16 of these 19 cases, there was client acceptance of the proposals. In line with the notion of preference, acceptances were typically delivered without delay in the simple form of the response token “Okay,” intoned with falling pitch; this accords with the analysis of patient acceptance of treatment recommendations in human medicine [34,35,36,40,47]. Among the 16 acceptance cases, 14 involved early client acceptance of the proposals, coming promptly on the heels of the veterinarians’ proposal turns. In two segments, there was delayed client acceptance because of constraints imposed by the linguistic design of veterinarians’ initial proposals. They were formatted as complex turns that did not provide a clear opportunity for the clients to respond and did not otherwise clearly expect initial client acceptance or declination because the proposal turns ended with only information-sharing by the veterinarian. In those two cases, the veterinarians treated client acceptance of their initial dietary proposals as due by pursuing (and ultimately receiving) client acceptance through the subsequent re-issuing of their proposals.

Three of the 19 cases lacking active resistance contained client responses other than acceptances. In one of these segments, signs of passive resistance to the dietary proposal were demonstrated by one of two clients attending a problem visit involving a dog with a cut paw. Passive resistance in conversation-analytic work in human healthcare encounters can involve silence and/or unmarked acknowledgment tokens (e.g., “Mm hm”) following the clinician’s health advice [43] or treatment recommendation. [36]. In another case, the client failed to respond to the veterinarian’s dietary proposal which had been uttered during other medical activities involving a client’s competing serious concern about the patient. The final segment was a case in which the veterinarian’s complex dietary proposal was designed in such a way that the status of the client’s response as acceptance was ambiguous. 

#### 2.5.2. Cases with Active Client Resistance 

The collection of 42 proposal segments included 23 (55%) segments (Figure 1) containing one or more instances of active resistance by clients in the form of asking questions or making statements that explicitly suggested various grounds for potential disaffiliation with the dietary proposals. A range of response types was identified in the 23 segments, such that some of the segments containing active client resistance opened with early acceptance or passive resistance to veterinarian’s dietary proposals before active resistance was displayed. 

## 3. Results

### 3.1. Characteristics of Appointments with/without Active Client Resistance

In order to explore whether the prevalence of clients’ active resistance varied depending on patient species and type of appointment, we drew on counts for those characteristics generated for our earlier study [52] (see Table 1). The number of appointments exceeds 35 because three appointments each contained two proposals, one of which elicited no active resistance and one of which elicited active resistance. Due to the unequal numbers of appointments associated with the presence versus absence of active resistance, prevalence figures are reported below as percentages to aid in making comparisons.

When we compared the proportions of appointment characteristics across the two sets of client response categories, there were some noteworthy differences (Table 1). Compared to the relatively even percentages of species in the overall collection of 35 appointments, in the 21 visits containing active resistance, cats were over-represented and dogs were under-represented. Conversely, in the 17 appointments in which clients displayed no active resistance, dogs were over-represented and cats under-represented. In terms of visit type in the overall collection of 35 appointments, frequencies were skewed, with more wellness than problem visits. When appointments containing active resistance cases were examined, the proportions between wellness and problem visits were slightly less skewed than in the overall collection and more skewed than in appointments lacking active client resistance. Cases with active client resistance tended to feature proportionally more cats and more problem visits than cases without active resistance (Table 1).

### 3.2. Analysis of Clients’ Active Resistance

#### 3.2.1. Key Features

Conversation analyses demonstrated that the display of clients’ active resistance was a complex, subtle interactional phenomenon. None of the actively resisting responses involved strongly oppositional, blatant rejections of veterinarians’ proposals. In accord with the conversation-analytic principle of preference, whereby recipients’ rejections of interlocutors’ proposals tend to be done as *dispreferred turn shapes* [69], clients’ active resistance of veterinarians’ proposals for long-term dietary modification was typically delayed and mitigated, sometimes dysfluent in its delivery, and consisted of or was often accompanied by elaborated accounts providing reasons for the possible or probable rejection of the proposals. Thus, active resistance did not position clients as uncooperative towards veterinarians’ advice in a blatant fashion. Indeed, some multi-unit turns in which clients demonstrated receptivity to veterinarians’ nutritional expertise (e.g., asking an advice-seeking question, accepting an offer of written information about a recommended prescription diet) were the same ones in which clients showed active resistance by orienting to possible barriers to successful adherence (e.g., Extract 5.1 below).

As mentioned previously, in non-resistance cases containing client acceptance, veterinarians’ dietary proposals needed to be linguistically designed as clear recommendations or suggestions for client responses to be unequivocally classified as acceptance. In contrast, client active resistance could come after clearly recognizable recommendations (e.g., “I would recommend you get him on at least partly canned food”) or more tentative utterances that were merely descriptive statements (e.g., “There are some excellent diets available for dogs…”) or evaluations (e.g., “One thing that’s really easy to do is…actually feeding a dental diet”; Extract 2.1 below). Clients oriented to veterinarians’ descriptions and evaluations as projecting future recommendations to change their pets’ diets by disclosing potential or actual obstacles to acceptance of the anticipated proposals. Veterinarians could thus test client receptivity to the possibility of dietary change without having to manage the interactional consequences of client disaffiliation with a canonical recommendation [52]. This was yet another manifestation in our corpus of the conversational preference for minimizing disagreement. 

As will be seen in the analysis of illustrative extracts below, client active resistance sometimes occurred after a display of initial acceptance (e.g., Extract 4.1) or passive resistance (e.g., Extract 3.1). In terms of how active resistance was demonstrated, clients asked questions or offered comments that flagged why the proposal or its adoption might be problematic in some way, proposed alternative actions that deviated from those recommended by the veterinarian, and/or shared information that provided accounts as to why the proposal was unnecessary or why the dietary change itself was irrelevant, ill-advised, or unfeasible. Because of the importance of such accounts in clinical understandings of nonadherence, we engaged in close investigation of the types and frequencies of the different grounds that clients gave for resisting veterinarians’ initial proposals for long-term nutritional change and the sequential environments in which they occurred.

#### 3.2.2. Grounds for Clients’ Active Resistance

Table 2 shows the types and prevalence of different categories of active resistance and examples of client utterances for each category in relation to the nutritional changes veterinarians had proposed. Across the 23 segments in which client active resistance was identified, there were 31 discrete client mentions of different bases for resisting long-term dietary change. The frequency of displays of active resistance was greater than the number of segments because in some segments clients oriented to more than one reason for possible nonadherence (e.g., Extracts 2.1 and 3.1; Table 2, Examples 2, 11). We identified a total of 11 categories of client-identified issues to which active resistance was tied. The four most frequent bases for possible nonadherence were as follows: the patient’s food preferences and/or dispreferences (7/31; 22.58%); multi-pet feeding management issues of various kinds (6/31; 19.35%); current use of an alternative strategy to address the health concern motivating the dietary proposal (5/31; 16.13%); and current utilization of the proposed diet (3/31; 9.68%) (Table 2). 

The different types of warrants for nonadherence can be conceptualized broadly in terms of whether they constructed the proposal as unnecessary or the nutritional modification as unnecessary, inappropriate, or impracticable. First, clients sometimes oriented to the proposal itself as unnecessary by reporting that they were already enacting the proposed regimen (e.g., Extract 1.1; Table 2, Example 6) or using another practice to address the identified health problem (Extract 2.1; Table 2, Example 5). In such cases, clients affiliated with the premise that the patient health concern identified by the veterinarian called for action but disaffiliated with the associated inference that such a strategy was not already in place.

Second, in other cases, the client’s resisting turn made available an inference that the dietary intervention was not relevant. This resistance was warranted in various ways. In two cases, clients questioned the veterinarian’s stance that the current food was likely contributing to the patient’s health problem (Table 2, Example 8). In Extract 3.1 below, a client challenges the veterinarian’s hypothesis that a food allergy might be contributing to the patient’s recurrent ear infections. In Extract 4.1 below, the client accepts that the dietary change is ultimately called for but justifies a delay in enacting the recommended food change by invoking expert information conflicting with the veterinarian’s proposal.

Third, other instances of client resistance suggested that the dietary change was not appropriate due to the presence or risk of additional health problems different from those triggering the proposal. Resistance was predicated on the basis that other medical conditions could be created or current ones could be exacerbated, if the dietary change were to occur. 

Sometimes the conflicting health issues were confined to a single patient and involved current comorbid medical conditions or the possible advent of a new, additional health concern if the food change targeting the identified health concern were to be adopted (Table 2, Example 7). In other instances, the competing health concern was associated with multi-pet feeding involving two or more pets (e.g., Extract 2.1). Active resistance oriented to the dangers of excess or inadequate nutrition (or both) associated with the proposed dietary modification. Such instances were situated explicitly or implicitly in the context of free feeding and/or shared feeding practices in multi-pet households. Clients’ accounts sometimes referenced food competition and differential feeding behaviors across pets in a household (e.g., intermittent feeding or “grazing” in one pet and rapid ingestion of an entire serving in another).

Fourth, dietary changes were sometimes resisted through client-generated accounts as to why it may not be feasible to enact the nutritional modifications despite their medical appropriateness. Clients described potential obstacles to adherence that were patient-based, client-based, or both. Patient-based accounts variously focused on pet preference for the currently favoured (to-be-eliminated) food (e.g., Extract 5.1; Table 2, Example 2) and/or dispreference for the proposed food (e.g., Extract 2.1; Table 2, Example 1); these accounts involved patients present in the consultation (Table 2, Example 1) or absent pets not part of the current visit (e.g., Extract 2.1). Dispreference-based accounts drew on specific past experiences with the proposed food or on a more general history of pet rejection of novel food items. In cases involving the proposed elimination of a favoured food, concerns about patient deprivation were sometimes raised (Table 2, Example 4). Other cases invoked multi-pet feeding management issues in the form of “dose”-related challenges: The successful implementation of the recommended nutritional change for a particular pet was characterized as uncertain or unlikely because of the difficulty in ensuring that this pet would actually follow a proposed regimen, such as reducing intake of dry food and increasing intake of canned food to manage weight in one patient in a multi-pet household (Table 2, Example 3). 

Assorted client-based barriers were described. In two cases, active resistance was conveyed through clients asking questions or making comments that oriented to the possible high cost of proposed foods (e.g., Extract 5.1; Table 2, Example 9). In another case, the client appealed to the veterinarian by projecting a loss of personal enjoyment should the hedonic feeding of a favourite treat to the patient be stopped (Table 2, Example 11). In a different case, resistance involved patient side effects described as aversive for the client (i.e., a history of patient flatulence associated with past food changes) (Table 2, Example 10). In a final case, the client told the veterinarian they lacked knowledge about the proposed food (Table 2, Example 12).

#### 3.2.3. Example Analyses of Clients’ Active Resistance 

Conversation analysis and its close attention to sequential organization showed these veterinarians’ proposals for long-term dietary change to be interactionally problematic. That is, the proposals were ill-fitted by definition in having produced clients’ dispreferred responses and accounts justifying non-acceptance. Our analyses provided important clues as to how veterinarians’ proposals might have been optimized and active client resistance potentially avoided or at least attenuated had certain topics of discussion between veterinarians and clients been introduced earlier and permitted to shape the ensuing conversation, including treatment decision-making. 

In this section, examples of analyses of veterinarian–client interactions featuring cases of client active resistance are provided. Presentation of the data in the form of extracts of transcripts is included as an inherent part of the validation process in conversation analysis. As experienced conversationalists, readers are invited to verify analytic claims by inspecting relevant features of excerpted portions of transcripts in which analytic claims are inductively grounded. 

For ease of reader comprehension, the number of transcription symbols displayed has been reduced. Initials at the left margin are used to identify the veterinarian (V), the client (C), and the patient (P) where appropriate. Single parentheses are placed around words that were not clearly audible and hence are best guesses as to what was said. Nonverbal activities and other information (e.g., when the patient is the recipient of a particular turn at talk) are indicated through the use of italicized words inside double parentheses. The onset of overlapping talk involving two speakers is represented by the use of square brackets. Curly brackets are used to anonymize a brand, product, or parent company name. The length of pauses or gaps in talk is captured through numerals to the tenth of a second, enclosed in single parentheses. Underlining demonstrates particular stress placed on a word or syllable, and extra letters signify the elongation of a word or syllable (sound stretching). Equal signs demonstrate latching, when there is no gap between individual words in a turn or between different turns at talk. The use of a question mark indicates rising pitch and a period following a syllable or word indicates falling intonation (final pitch). Degree signs around a word or syllable show that this talk is noticeably quieter than the surrounding talk. Bold font signals those stretches of clients’ talk constituting active resistance and/or reasons given for resisting veterinarians’ proposals to modify patients’ diets. 

In Case 1, the client resists the dietary proposal on the grounds that she is already feeding the recommended diet. Extract 1.1 shows initial passive client resistance followed by active resistance. This wellness visit involves a 13-year old female dog diagnosed with arthritis. Prior to the extract below, the client reported to the veterinarian that the patient was very stiff with arthritis. The veterinarian discussed arthritis medications and the client expressed an interest in trying an arthritis medication to see if it would help the patient’s mobility. The extract begins with the veterinarian announcing the results of the weigh-in of the patient (line 1).



**Extract 1.1.**


1

 V: Uh she’s ninety-one pounds but that’s [exactly

2

 C:                            [Okay

3

 V: what she she’s exactly what she was last year=

4

 C: =(Yeaaaah.)

5

    I thought she was. Yeaah.=

6

 V: =And [and that’s

7

 C:      [Yeah.

8

 V: that’s good. I mean 

9

 C: [(Yeah.)

10

V: [One of the problems that we do run into

11

   with arthritic dogs is that because they exercise less

12

   they tend to gain weight.

13

C: Mm hm=

14

V: =And every extra pound that they’re they’re weighing

15

   is more pressure

16

C: [Yeah.

17

V: [on those arthritic joints.

18

   So.

19

C: Yeah.

20

V: You know i i uh just mentally

21

   you should make a note of that and say you know=

22

C: =Mm hm.

23

V: Maybe she doesn’t need as much food as she used to.

24

C: Mm hm.

25

V: Or maybe it’s time to switch to a senior food

26

   or a light food

27

C: [Yeaah.

28

V: [°It probably makes° it probably is time to switch

29

   to a senior food

30

C: Yeaah. **[she is**

31

V:        [You know if she’s [thirteen years old.

32

C:                            [Yeah.

33

V: Yeah it’s about time. (Is it about time?) *((to P))*

34

C: Yeah I know. I actually have her on that sooo




Of analytic import is the design of the veterinarian’s turn on lines 1 and 3. Using the contrastive conjunction “but,” the weight announcement is tied to the subsequent statement, “but she’s exactly what she was last year.” “But” serves to somewhat downgrade the newsworthiness of the weight announcement; it also packages “ninety-one pounds” in the reassuring context of ongoing weight maintenance rather than as new weight gain. This move is perceived as a bit of a mixed message; in presenting patient weight as requiring such reassurance, the veterinarian’s comments make available an inference that the patient’s weight is problematic in some way.

On line 2, the client’s “Okay” response acknowledges the information about the patient’s current weight as identical to last year’s. The client’s comment, “I thought she was,” and affiliative “Yeaaaaah” (line 5) orient the information as positive and position the client as a pet owner who has been successfully monitoring and maintaining the dog’s weight over the past year. That the lack of change is laudatory is indicated by the veterinarian’s subsequent positive assessment (“that’s good,” line 8), with which the client agrees (line 9); however, the veterinarian’s “I mean” (line 8) signposts a possible adjustment to this positive evaluation [70]. This comes in the form of expert information-sharing warning about the increasing risk of weight gain in arthritic dogs due to their restricted mobility (lines 10–12) and the subsequent negative effect on their diseased joints (lines 14, 15, 17). “So” (line 18) projects the veterinarian’s subsequent delivery of the upshot of this health risk [71] in the form of a mitigated recommendation that the client consider reducing the patient’s food intake: “You should make a note of that and say you know maybe she doesn’t need as much food as she used to” (lines 21, 23). 

On line 24, instead of uttering “Okay,” which in this slot would perform the acceptance of the recommendation, the client responds with “Mm hm”; this constitutes passive resistance in its dual function as an acknowledgment token and continuer supporting the veterinarian’s continuing possession of the conversational floor [72]. In the absence of the client’s acceptance of the recommendation, the veterinarian issues a different proposal, this one involving a change in food choice to either a senior or light formulation (lines 25, 26). The “or” preface at the start of line 25 latches this multi-alternative proposal to the prior, rejected proposal, packaging all three recommendations as options. This design downgrades the prescriptiveness of the deontic stance of the veterinarian and simultaneously minimizes the appearance of client disagreement by obfuscating the client’s lack of affiliation with the veterinarian’s prior suggestion to reduce food intake. 

Of analytic interest in this extract are the client’s “Yeah” turns, uttered with final pitch (lines 16, 19, 27) as response tokens during the veterinarian’s description of the health risks and associated dietary proposals. While these tokens enact the listener role of the client in acknowledging the information carried by the veterinarian’s contributions and acting as continuers supporting the veterinarian’s ongoing role as speaker, “Yeah” uttered with falling intonation is more oriented to the potential speakership of its user and the level of her involvement in the conversation than “Mm hm” would be in those slots [72]; the implication is that the client may well have something substantial to add to the discussion. The client’s “Yeah” (line 27) overlaps with the veterinarian’s proposal (the line to switch the patient to a senior formulation; lines 28, 29). The “yeah” preface on line 30 begins what is perceived as the start of the client’s actively resisting utterance. It is self-aborted, dropping out after “she is” is spoken in overlap with the veterinarian’s justification for changing to a senior formulation on the basis of patient age (line 31). 

After the veterinarian recycles part of the proposal, seemingly while addressing the patient (line 33), the client finally produces and completes the actively resistant turn in the clear: “Yeah I know. I actually have her on it that” (line 34); “I know” treats the proposal and the reasoning accompanying it as unnecessary by underscoring the epistemic stance of the client as knowledgeable about the health risks to the patient and of the appropriate remedy; “actually” indexes the client’s resisting utterance as a *counter-informing* [73] that corrects the veterinarian’s preceding presupposition that the client was not feeding the patient a senior formulation. At issue here are the client’s epistemic primacy regarding intimate knowledge of the current feeding practices for this pet and the veterinarian’s corresponding lack of knowledge. 

Extract 2.1 depicts Case 2 in which a client actively resists the dietary proposal on multiple grounds: that the patient has previously rejected the recommended food; that another pet in the home is at risk of developing health problems were the food to be re-introduced; and that the client is using an alternative strategy to address the health problem the proposed dietary change was intended to address. The client displays passive resistance and then active resistance to the veterinarian’s proposal. The extract is taken from a wellness appointment with a male cat. Prior to the extract, the veterinarian informed the client after the oral examination that there was tartar on the patient’s teeth, discussing dental scaling first and then the idea of training the patient to have his teeth wiped or brushed as a means of slowing down tartar build-up and delaying the need for the dental procedure. The client laughed and said “Good luck,” invoking patient noncompliance to resist the veterinarian’s proposal to brush the patient’s teeth. 



**Extract 2.1.**


1

  V: Uh one thing that’s really easy ta do

2

    (0.5)

3

    not as effective is is actually feeding a dental diet.

4

    (0.2)

5

  C: Hmm *((Nods head))*

7

  V: A dental [diet’s

8

  C:          **[I did give it to him but he um**

9

     **[he**

10

 V: [He didn’t like it?

11

 C: **He didn’t really like it [much**

12

 V:                              [(No?)

13

 C: **But the-deh his sister Tidbit.**

14

 V: Loved it too mu[ch? Huh ha-ha=

15

 C:                [Ohh God

16

 V: =ha-[ha

17

 C:     **[Well she’s now fifteen pounds**

18

   **and she just turned a year.**

19

   [°So.°

20

 V: [Uuhkaay

    (0.4)

21

 C: **Eats everything in sight.**

22

 V: °’Kay.°

23

   There are other things that can be done.

24

    [There are solutions you can=

25

 C: [**But he gets I give him dental treats.**

26

     V: =add to the water?




The veterinarian’s proposal on lines 1 and 3 is designed as a description of an alternative strategy, “feeding a dental diet,” which has embedded in it assessments that characterize this dietary approach as “really easy” but not as “effective” as tooth brushing. The assessment of a dental diet as “easy” constructs it as advice-implicative [74] and contrasts it with the difficulty of attempting to brush the cat’s teeth, something the client previously indicated. The informative features of the dietary proposal (lines 1, 3) implicitly position the client as someone who has never used a dental diet and explicitly construct the diet as an easy strategy to adopt.

Both of these aspects turn out to be problematic from the client’s perspective. The client responds initially with the minimal response token (“Hmm”) and head nod (line 5) that together merely acknowledge receipt of this information. Because this proposal is designed to present one option among several rather than as a singularly preferred course of action, the client’s response is a milder form of passive resistance than might be called for if the veterinarian had said “I would recommend you put him on a dental diet.” The veterinarian starts providing more information about dental diets (line 7), at which point the client, in partial overlap with the veterinarian, begins an actively resisting turn (line 8). In contrast to the canonical form of acceptance in our data (a prompt “Okay” with falling pitch), the client’s response here displays the features of a dispreferred turn shape. It is performed with hesitation and disfluency: “I did give it to him but he um he” (lines 8, 9). The “but” clearly forecasts a problem with the dietary proposal, which the client seems reluctant to share.

The hitches and perturbations in the client’s talk threaten the progressivity of the discussion. The veterinarian assists on line 10 as part of a *collaborative turn sequence* [75]; the veterinarian reacts to the trouble spot in the conversation with a contribution that completes the client’s turn and hence affiliates with the client’s experiences. Using a yes/no type declarative question [76], the veterinarian anticipates what the client was going to say and proffers a possible candidate completion of the client’s turn for the client to accept or reject: “He didn’t like it?” (line 10). In posing this as a question, the veterinarian ratifies the epistemic primacy of the client’s knowledge of the patient’s food preferences while still being the first person in the encounter to actually suggest the specific obstacle to adherence: the patient’s dislike for the recommended food. In displaying knowledge about the common challenges of cat ownership, even though lacking knowledge about this specific patient, the veterinarian bridges the earlier misalignment with the client created by the ill-fitting proposal. The client accepts the candidate completion as their own by recycling it, slightly downgrading the extent of the patient’s aversion to dental food by incorporating “[not] really” and “much” (line 11). In doing so, the client reduces the degree of disaffiliation with the proposal and reasserts their superior epistemic status as the expert on their pet’s food preferences. This status is upheld by the questioning “No?” on line 12, which positions the veterinarian as less knowledgeable on the matter of the patient’s feeding history and preferences than the client.

The client then uses the disjunctive “but” to contrast the patient’s evaluative stance toward dental food with that of a female cat in the household (line 13) not present at the appointment. This launches the start of yet another reason for resisting the proposal: the potential negative health consequences for another cat in the household. Once again, the veterinarian uses a collaborative turn sequence, offering another yes/no declarative question for client ratification in the form of a different barrier to adherence posed by the other cat overindulging in the dental diet (line 14), the implications of which are softened by trailed-off laughter (lines 14, 16). The client begins a response to the candidate completion with “Oh God” (line 15), an emotionally charged, nonconforming response. The “oh” preface here establishes that the client has greater epistemic authority than the veterinarian regarding claims about the absent Tidbit, in light of the client’s primary epistemic access to information about her [63].

The client then delivers an answer to the veterinarian’s question starting with a “well” preface (line 17). This feature flags that the answer will be an expanded response, not a straightforward agreement [77] with the veterinarian’s suggestion that Tidbit merely loved the dental diet “too much.” The client’s announcement of Tidbit’s current weight and young age (lines 17, 18) makes available a new inference about a risk of lifetime overweight. The low-volume “so” (line 19) leaves implicit the upshot of the prior talk about Tidbit’s health issues: that a dental diet is contraindicated. The client’s information-sharing about Tidbit is received with a marked acknowledgment (line 20) that shows the veterinarian to accept the veracity of the health risks to her. The severity of the risk posed to Tidbit by hypothetical reintroduction of a dental diet is further underscored by the client’s description of Tidbit as a cat that “eats everything in sight” (line 21), a fact that the veterinarian quietly acknowledges (line 22). “Everything” makes this an extreme case formulation [78] that by definition is rhetorically designed to discourage the undermining or challenging of the client’s active resistance by the veterinarian.

Abandoning the dental diet option, the veterinarian counters the apparent hopelessness of the situation with a generic announcement about “other things that can be done” (line 23). Partway through the veterinarian’s description of solutions that can be added to the drinking water, the client actively resists this strategy by informing the veterinarian of an additional, heretofore undisclosed, strategy already adopted for addressing dental concerns: the use of dental treats (line 25).

In Case 3, a client questions the veterinarian’s conjecture that the patient’s health problem could be caused by the current food he is being fed. Extract 3.1 contains an instance of initial client passive resistance followed by active resistance. This is a problem visit involving a dog who has a history of ear infections since puppyhood and has recently begun repeatedly scratching one ear and whimpering. Prior to the extract, the veterinarian asked the clients what food the patient was on. They described a lamb and rice calorie-reduced formulation the patient had been eating since coming to their home from the breeder. The veterinarian justified asking about diet because of a possible link between food and recurrent infection. Information-sharing about both food allergies and a possible thyroid insufficiency, raised because of the patient’s age, ensued. Right before the start of Extract 3.1, the veterinarian indicated that a thyroid problem, while possible, was less likely than food allergies.



**Extract 3.1.**


1

 V:  Food issues

2

     (0.5)

3

     Possible

4

 C1: Yeah. Yeah.

5

 V:  You know and one thing ummm you may wanna consider

6

     is trying him on a vegetarian food

7

 C1: Mm hm. *((C2 nods))*

8

 V:  Um if you wanna pick it up a-at the pet store

9

     {Parent Company} has one *((C2 nods))*

10

    that seems to be very good.

11

C1: Mm hm.

12

V:  Um the prescription hypoallergenic foods are

13

    (0.3) better?

14

C1: Yeah

15

V:  From a (0.3) formulation standpoint but

16

C1: Mm hm.

17

V:  It’s something you know to think about.

18

    (0.3)

19

C1: [Yeah

20

V:  [You know cuz it could certainly be related to

21

    (0.3) the food

22

V:  Hi baby *((to P))*

23

    (1.0)

24

C1: **Or just the fact that it’s such a**

25

    (0.9)

26

    **dark damp mess in there**




The proposal to consider trying vegetarian food (lines 5, 6) comes immediately after the veterinarian’s listing of “food issues” (line 1) as a hypothetical contributor to the patient’s ear infection (line 3). Instead of accepting the proposal with “Okay,” Client 2 demonstrates passive resistance by responding on line 7 with the continuer “Mm hm,” a stance further exemplified by Client 1’s silent head nod (line 7). The veterinarian pursues more engagement from the clients, keeping the topic alive by first offering a product name and assessment of a high-quality vegetarian food available at a pet food store (lines 8–10), which Client 1 acknowledges (line 11). The veterinarian then provides an assessment of in-clinic prescription diets as better than store-bought, to which Client 1 responds with “Yeah” (line 14). The veterinarian continues the topic with a turn increment (line 15) that receives minimal acknowledgment from Client 1 (line 16).

The ending of the sequence is forecasted by the veterinarian’s recycling of the proposed diet as “something you know to think about” (line 17); this receives no client acceptance in the form of an “Okay” but the more ambiguous and slightly delayed “Yeah” (line 19) in overlap with the veterinarian’s conjecture that the ear infection “could certainly” be related to the patient’s current diet (lines 20, 21). This conjecture is an upgrading of epistemic confidence from line 3 where a food allergy was assessed merely as a “possible” contributor to the current medical problem. The veterinarian then addresses the patient (line 22) on the examining table.

It is at this late point in the segment that Client 1 demonstrates active resistance. After a 1.0 s delay, the client’s “or” preface links this resisting turn to the veterinarian’s prior conjecture (lines 20, 21) by proposing an alternative explanation for the patient’s ear infection, one that subtly challenges the food allergy hypothesis: “Or just the fact that it’s such a (0.9) dark damp mess in there” (lines 24–26). The gap in talk points to the hesitancy with which this challenge of the veterinarian’s expertise is conveyed: that the structure of the patient’s ear makes it a natural site for infection. While the “or” preface, on the one hand, retains this proposal’s status as a conjecture that does not trump the veterinarian’s prior one, “just” on the other hand marks the client’s hypothesis as the more parsimonious explanation and “fact” promotes its incontestability.

Case 4 contains an example of initial client acceptance of the veterinarian’s proposal followed by active resistance. Extract 4.1 is taken from a wellness visit involving a 17-month old female Labrador retriever. Prior to the extract, the client answered the veterinarian’s diet history question by indicating that the patient was on a higher-quality grocery store brand of puppy food mixed with canned food. This segment occurred partially off camera, with the veterinarian and client on the floor with the patient. On line 1, the veterinarian focuses on the age of the patient as noteworthy by issuing a yes/no type declarative question asking the client to confirm the candidate estimate of the patient’s age. In asking this question in the context of not having the patient file available during the examination, the veterinarian acknowledges the client’s superior epistemic status regarding knowledge of the patient’s age.



**Extract 4.1.**


1

  V: Now (0.4) she’s like a year and a half right?

2

  C: One year five months. The twenty [fourth.

3

  V:                                  [Yeah.

4

  C: November [twenty fourth

5

  V:          [Soooo

6

     she should be gettin’ onto adult food.

7

  C: Okay. 

8

     (0.2)*((Clapping sound))*

9

  C: **We’ll finish this ba[g? and she’ll go on=**

10

 V:                        [Yeah. Like

11

 C: **=[adult food**

12

 V: [switch her over to adult

13

 C: ‘Kay?=

14

 V: =Uumm

15

    (0.5)

16

 C: **Cuz it says up to two years on the bag**




A type-conforming response to the veterinarian’s question would be “yes” or “no.” The client’s answer (lines 1, 2), structured as nonconforming and dispreferred, treats the veterinarian’s question as problematic in some way [76]. The answer invokes the client’s epistemic primacy, as a pet owner with greater familiarity about the pet’s biography than the veterinarian, to correct the imprecision of the veterinarian’s over-estimation of the patient’s age. This interactional detail prefigures the nature of the active resistance that subsequently emerges. The veterinarian’s “Like a year and a half” (line 1) becomes “One year five months” (line 2) in the client’s answer, which is expanded to include an announcement of the specific date on which the patient turned this age (lines 2, 4). The veterinarian acknowledges the initial part of the client’s response (line 3) and, in overlap with the expanded answer (line 4), delivers an elongated “so” preface [70] (line 5) that displays the upshot of the previous exchange about the patient’s age: the need to switch to adult food (line 6). This proposal is carried out in a relatively prescriptive fashion using the verb of necessity “should” [52].

The client initially accepts the proposal using the canonical response token “Okay” delivered with final intonation (line 7). The client expands their acceptance turn by announcing their own counterproposal: to finish the current bag of puppy food before introducing adult food (lines 9, 11). The veterinarian responds in such a way as to treat the client’s plan as a problem. The turn-initial “Yeah” (line 10) acknowledges the client’s proposal but does not accept it in the way that a falling-pitch “Okay” would have; the veterinarian recycles the proposal (lines 10, 12), which focuses exclusively on the food switch and deletes any reference to the client’s proposal to use up the current bag of puppy food. The client’s contracted “Okay” on line 13 has rising intonation which renders it ambiguous with respect to its function as acceptance of the re-issued dietary proposal; the veterinarian comes in immediately with a sound-stretched “Um” (line 14) which appears designed to reserve the conversational floor on their own behalf while simultaneously signalling a significant delay in speakership [79] (line 15). However, half a second later, which is a fairly lengthy gap in conversation [80], the client seizes the floor, giving a justification for their proposal to delay the food switch (line 16): “Cuz” makes explicit the status of this utterance as an account for the now clearly problematic proposal to delay the food switch. Here, active resistance comes in the form of citing the feeding instructions on the bag of puppy food giving two years as the upper age limit, information that is in conflict with the recommendation of the veterinarian. Client active resistance in this example is grounded in an epistemic clash between two conflicting sources of expert knowledge: the veterinarian and the pet food company that manufactured the current food.

In Case 5, active resistance is subtle, a kind of implicature in its mention of the possible expense of the recommended food and a comment about the food preference of another cat in the household. Extract 5.1 comes from a wellness visit with a male cat recently adopted from the Humane Society. Prior to the extract, the veterinarian asked history-taking questions, including one about the type of main food in the patient’s diet, and completed an oral examination. The veterinarian announced to the client that the patient had some gingivitis, tartar, and plaque on his teeth, showing the client the evidence. The veterinarian explained that the best way to avoid this is to brush the patient’s teeth daily, something that can be difficult to perform with cats. The non-adoption of the tooth brushing option was normalized by the veterinarian whose own cat was “not nice enough” to permit it.



**Extract 5.1.**


1

 V: The other option would be to have him on a special

2

     um dental diet. *((C nods))*

3

    Uh the dental diet that I recommend is the 

4

     {2 Brand-Relevant Initials} diet it’s for 

5

    [tooth diet

6

 C: [°Mm.°

7

 V: It’s made by {Pet Food Company Name} and it’s special

8

    because the kibbles are larger *((C nods))*

9

    than other kibble? and the tooth has to go all the way

10

   into the kibble before it shatters. So it acts like a

11

   toothbrush brushing up on the teeth when they chew the 

12

   food.

13

C: °Okay.°

14

V: Uh the last option would be to just wait until the 

15

   teeth get bad enough and then do a dental cleaning 

16

   and with cats with a dental cleaning it’s anywhere 

17

   from two hundred and fifty dollars to about

18

   [three hundred and fifty dollars.

19

C: [°Hm° 

20

V: And it is under general anesthetic so it is a big

21

   deal.

22

C: Mm hm.

23

V: So it’s kinda nice to try to avoid that if you can.=

24

C: =(Yeah.)

25

V: So if you’re at all interested in the diet that I was

26

   talking about I can definitely send you home with a

27

   pamphlet?

28

C: That’d be good **(give us) a price range [on it=**

29

V:                                            [Yup

30

C: **=(°kind of thing.°)** *((C nods))*

31

V: And then if you decide you want it I can also send you

32

   home with a free bag to try just to see if he likes it

33

   and to see if you like it.

34

C: Yeah cuz Rascal’s been eatin’ hard kibble=Yeah **he**

35

   **likes the stuff he has**.




On lines 1 and 2, the veterinarian uses a description-type format to propose an option in lieu of tooth brushing. This initial proposal bears some resemblance to that appearing in Extract 3.1 (lines 1, 3), though that proposal was more strongly advice-implicative because it characterized the option as “easy.” In Extract 5.1, the client does not respond vocally but rather minimally displays their status as an attentive listener by nodding their head. The veterinarian continues sharing information about the name of the in-clinic product that they typically recommend (lines 3–5); this recommendation is designed as a generic one associated with typical advice-giving by the veterinarian (“that I recommend”), rather than as a recommendation targeting this client and patient specifically. This construction orients to the possibility that this product will not be appropriate for this client and/or patient. It manages possible resistance by allowing the veterinarian to “test the waters” with the client to gauge possible interest in the in-clinic dental diet. The client demonstrates very limited engagement, quietly using the weak acknowledging token, “Mm” (line 6) [81]. The veterinarian shares more information about the parent company and design of the dental kibble (lines 7–12), which the client acknowledges in a more marked but still quietly uttered way with “Okay.” Although the client’s responses might appear at first blush to display passive resistance, it should be noted that none of the veterinarian’s contributions thus far are designed as a clear recommendation targeting the patient, but are rather packaged as information. Hence, client acceptance or rejection is not expected in the way that “I would recommend you have him on a dental diet” would have.

The veterinarian then describes the final treatment option of dental scaling (lines 14, 15), providing a price range (lines 16–18) and an assessment (lines 20, 21), underscoring the seriousness of an intervention involving general anesthesia, which the client receives with “Mm hm” (line 22). The veterinarian then produces a “so”-prefaced assessment (line 23), signalling the logic involved in adopting a dietary approach: This assessment is advice-implicative in that it implicitly re-introduces the dental diet as a means of avoiding the cost and health risks to the patient posed by dental cleaning. The client appears to affiliate with this assessment by uttering “Yeah” (line 24). The veterinarian then offers a pamphlet containing information about the in-clinic dental diet (lines 25–27); the use of the if/then clausal structure delicately manages any inferences about sales pressure by making it clear that this offer is contingent on the client’s interest, the presumption of which is minimized through the use of “at all” (line 25). Thus, a decision to purchase the proposed food is deferred to an uncertain future.

After initially accepting the offer with a positive assessment on line 28, the client then displays active resistance related to the expense of the dental diet by soliciting a price range (line 28, 30), something the veterinarian immediately promises to provide (line 29). Beginning on line 31, the veterinarian then delivers a second offer of a free sample of the food to “try,” constructed using “if”-prefaced clauses that orient to contingencies in the form of possible dispreference for the product on the parts of the patient (line 32) and client (line 33). The client does not accept the offer; instead of using “Okay,” they use “Yeah” to affiliate with the concern the veterinarian raises about food preference as another possible obstacle to the adoption of the new food: the possible rejection of the new food by a different cat in the household not in attendance at the visit (lines 34, 35).

In sum, conversation analysis of clients’ initial active resistance to veterinarians’ proposals for dietary change underscored the importance and dynamics of the epistemic states, statuses, and stances of veterinarians and clients. As illustrated above, nutritional proposals were sometimes issued before veterinarians had educated the client about nutrition and/or the relevant current diagnosis or potential health problem that the proposed change was intended to address or prevent; in such cases, clients sometimes questioned or challenged the relevance of the dietary proposal because they did not understand the link between the current diet and health problems. In other cases, because veterinarians had not previously conducted a thorough nutritional assessment, including dietary history-taking, they were unaware that the patient was currently on the recommended food or that it had been tried, unsuccessfully, in the past. In the latter case, this meant that the veterinarian lacked knowledge of key client-related or patient-related obstacles to adherence. And while the proposed dietary change was sometimes situated within a larger set of treatment options, including non-nutritional strategies, analyses of our collection showed that veterinarians did not explicitly solicit clients’ perspectives and possible concerns about treatment options, dietary or non-dietary, before delivering their proposals. Therefore, prior to proposing modifications, some veterinarians lacked knowledge about patient or client preferences and client worries about matters such as expense and undesirable consequences of nutritional change.

### 3.3. Analysis of Veterinarians’ Uptake and Interactional Outcomes

#### 3.3.1. Key Features

This section reports on patterns of interaction and decision-related outcomes in the wake of clients’ active resistance. Analyses highlight veterinarians’ uptake, the ensuing client–veterinarian interactions, and what sorts of treatment plans, if any, were agreed upon by the end of the appointment. Veterinarians’ responses to clients’ active resistance were complex and variable, demonstrating the dynamic epistemic and deontic territories they occupied.

In epistemic terms, veterinarians’ responses often contained recommendation-relevant information-sharing, including their medical justifications for the proposals. A total of 16 of the 23 resistance cases (69.6%) involved recycling of the initial dietary proposals, often in modified form, which sometimes closed down resistance-related talk and dietary discussion more generally. Veterinarians sometimes oriented to prior displays of client resistance by downgrading the proposal to a mere consideration of the dietary change or replaced the original proposal with an alternative dietary or non-dietary strategy for addressing or preventing the health issue (e.g., Extract 2.2 below). Thus, veterinarians exemplified their deontic right to recommend actions in the service of patient health but mitigated their deontic authority in imposing particular actions on clients and patients.

Table 3 shows the final outcomes of decision-making trajectories following active client resistance. Among the 23 active resistance cases, 10 cases (43.5%) involved the client ultimately accepting an alternative strategy or strategies to manage the health concern; this occurred after the veterinarian’s original dietary proposal was abandoned in the wake of client resistance. The agreed-upon strategies could be nutritional or non-nutritional, including generic, downgraded recommendations that clients simply monitor the health issue (e.g., Extract 1.2). Thus, even in cases in which veterinarians accepted the basis for client resistance, they did not necessarily do so in a manner that concomitantly downgraded the seriousness of the identified health concern. In one of these cases, the veterinarian flagged that further consideration of the proposed dietary change could happen at a subsequent appointment at which the agreed-upon alternative strategy for addressing the health issue (anal gland expression) was scheduled.

Eight cases (34.8%) resulted in client acceptance of the veterinarian’s initial nutritional proposal (e.g., Extracts 4.2 and 5.2) (Table 3). In four of the eight acceptance cases, client acceptance was *unmarked*, consisting of a falling-pitch “Okay” response without delay or elaboration. In conversation-analytic terms, the design of these turns as minimal and immediate showed them to be canonical preferred responses to veterinarians’ proposals. In the remaining four cases, client acceptance was *marked*: such accepting responses included additional lexical material suggestive of intensified client engagement. For example, in Extract 5.2 below, the client accepts the veterinarian’s offer of a free sample bag of dental diet and, as a way of addressing the previously identified potential barriers to adherence, proposes mixing the new food with the less expensive, favoured current food. Of note, here is how these results compared with our preliminary analysis of the non-resistance cases. In that collection, only two of the 16 early acceptance cases contained client responses that were more expansive than “Okay.”

In two cases (8.7%), the veterinarian postponed further discussion of dietary change, in one case until test results were available and in the other, as previously described, until a follow-up appointment (Table 3). In two other cases (8.7%), veterinarians accepted modified counterproposals suggested by clients. In a final case (4.3%), it was not clear by the end of the appointment as to whether clients had accepted the veterinarian’s dietary proposal because the veterinarian responded to the resistance with medical information-sharing that did not expect acceptance or rejection in the way that a clearly designed recommendation would have (Table 3).

#### 3.3.2. Example Analyses of Veterinarians’ Uptake of Client Active Resistance

In this section, we present additional conversation analyses of the five cases previously featured, with particular attention on veterinarians’ uptake of active client resistance and subsequent veterinarian–client interactions. For each case, we also provide a brief description of any further proposal-relevant talk occurring during the visit after the resistance segment ended.

Further analysis of Case 1 shows how the progress of a treatment conversation can become hampered when the veterinarian is unaware of what the client is currently doing to address a health problem. It also shows how the recycling of previous recommendations not well fitted to the particular client and patient can lead to further disaffiliation by the client. In this case, the veterinarian ultimately dropped the dietary proposal.

Extract 1.2 captures what unfolded after the client informed the veterinarian that the patient is already eating the senior food previously recommended (line 34).



**Extract 1.2.**


34

C: **Yeah I know. I actually have her on that sooo**

35

V: Okay.

36

C: Yeah.

37

V: All right. 

38

   So.

39

C: (But yeah.)

40

V: But just you know make a mental note that you won’t

41

   let her get overweight.

42

C: Yeah [yeah. I thought sh-

43

V:      [Cuz it’s it just gets

44

   harder and harder.

45

C: Oh yeaah. Exactly.

46

V: (Yeah)

47

C: I thought she was maybe about the same.

48

V: [She is.

49

C: [I didn’t notice 

50

   that she sort of bulked up any so

51

V: No=no she’s good.

52

   Hey sweetheart ((to P))

53

C: Oh good.




The accountability of the veterinarian in having delivered unnecessary dietary advice is conveyed by the elongated, trail-offed “sooo” (line 34) produced by the client at the end of the resisting turn; it acts as a prompt to the veterinarian to produce a relevant next action [71]. The veterinarian accepts the client’s informing with “Okay” (line 35); the client’s “Yeah” with falling pitch (line 36) appears to reaffirm their own earlier claim on line 34. The veterinarian’s “All right” (line 37) and stand-alone “So” (line 38) are potential change-of-activity tokens [71,82], signalling a possible shutting down of the sequence. However, the veterinarian next recycles one of their previous recommendations using the imperative form (lines 40, 41). The turn-initial “but” (line 40) marks this general advice to the client—to monitor the patient and avoid letting her gain weight in the future—as disjunctive with the client’s current strategy of using a senior food as a weight-control measure.

The client begins what is projectable as yet another actively resisting turn on line 42, issuing what appears to be a reiteration of their utterance on line 5 in Extract 1.1, stating their own independent perception that the patient had not gained weight over the past year. However, the client aborts completion of this utterance, dropping out in overlap with the veterinarian who justifies the recommendation with reference to their expert knowledge of the progressive challenges in keeping elderly, arthritic dogs from gaining weight: “Cuz it’s it just gets harder and harder” (lines 43, 44). “Exactly” in the client’s response on line 45 demonstrates strong agreement as a pet owner with the veterinarian’s assessment; in combination with its “oh” preface, this response positions the client as having independently and previously come to the same opinion as the veterinarian [83]. In claiming prior awareness of the increasing difficulties in preventing the dog’s weight gain, the client implies that the veterinarian’s explanation and advice are not needed.

On line 47, the client restarts and completes the utterance begun on line 42, which recycles the claim on line 5 in Extract 1.1: “I thought she was maybe about the same.” This repeat emphasizes the successful monitoring of the dog’s weight before attending the appointment, which suggests that the veterinarian’s injunction to avoid weight gain is unnecessary. The veterinarian agrees with the client’s perception of the patient’s weight (“She is”; line 48) in overlap with the client’s continued turn. The client’s statement that they “didn’t notice that she sort of bulked up” (line 50) results in the veterinarian’s reassuring assessment, “No = no she’s good” (line 51), which repeats the positive evaluation of the patient’s weight from Extract 1.1. The client receives the veterinarian’s assessment on line 53 with “Oh good.” Here, “Oh” marks the veterinarian’s assessment as news for the client [84]; in doing so, the client upholds the epistemic superiority of the veterinarian as the medical expert who determines what an acceptable weight for the patient would be. This subordination of the client’s own epistemic status on line 53 tempers the disaffiliative character of the prior talk. Ultimately, to support the mobility of the patient and encourage more exercise, the client later requests the pain medication for the patient recommended by the veterinarian near the beginning of the visit.

Case 2 is another case in which the veterinarian abandoned the original nutritional proposal. Extract 2.2 shows the veterinarian’s uptake of the first types of active resistance disclosed by the client in Extract 2.1: the patient’s dislike of the recommended dental food, which the veterinarian did not know the client had already tried using, and the risk of inducing overweight in another cat in the home, if the dental food were to be re-introduced.



**Extract 2.2.**


23

  There are other things that can be done.

24

  [There are solutions you can=

25

C: [**But he gets I give him dental treats.**

26

    V: =add to the water?

27

C: (Mm hm)

28

V: They’re also helpful

29

   and even (0.3) not even brushing the teeth 

30

   but just rubbing *((Demonstrates on P))*

31

   the gums up against the teeth? *((C nods head))*

32

   tha-that’s helpful [too at slowing down= 

33

C:                     [Ohh yeah? *((C nods))*

34

V: =dental tartar 

35

C: That’s okay honey. He’s just pokin’ ya *((To P))*




The veterinarian drops the food proposal, suggesting an alternative intervention (lines 24, 26). The client pre-emptively resists the veterinarian’s new proposal by announcing an alternative current strategy of which the veterinarian appears unaware: dental treats (line 25). The veterinarian responds to this with a positive assessment (line 28), and then, after discarding the previously rejected idea of teeth brushing (line 29), offers additional information about a different strategy, rubbing the patient’s gums against his teeth (lines 30–31). The “Oh yeah?” (line 33) treats this advice as new information to which the client appears receptive; however, the client’s subsequent talk to the patient (line 35) orients to the cat’s lack of cooperation with the veterinarian’s gum-rubbing (see [49,50]) and hints at the potential lack of future success in its adoption at home. It should be noted that the veterinarian does not take up this possible issue with the client. By assessing the client’s current strategy of dental treats as “also helpful” (line 28) and then appending the gum-rubbing strategy as “helpful too” (line 32), the veterinarian demonstrates support of the use of dental treats while implying that singular action (i.e., the current status quo) is not sufficient in and of itself. Thus, the veterinarian promotes a multi-pronged approach, justified in terms of delaying tartar accumulation (lines 34, 36).

In Case 3, the veterinarian dropped the proposal to switch to a vegetarian diet after the client’s suggestion that the patient’s recurrent ear infections had a different, non-dietary etiology. Extract 3.2 begins with the client’s hypothesis that the infections are caused by moisture trapped in the patient’s ears.



**Extract 3.2.**


24

C1: **Or just the fact that it’s such a**

25

    (0.9)

26

    **dark damp mess in there**

27

V:  Welll I mean he does have floppy ears

28

    I mean it does get hot and moist

29

    0.5 ((V sniffs P’s ear))

30

V:  And this is yeast




The veterinarian takes up the client’s active resistance of the conjecture on lines 24 and 26 with an answer that constitutes neither direct agreement or disagreement; the elongated “well” preface (line 27) projects that the veterinarian’s response will not be straightforward or concise. The two instances of “I mean” (lines 27, 28) signpost the veterinarian’s subsequent concessions [70] to the client’s theory by acknowledging that the “hot and moist” (line 28) environment engendered by the patient’s “floppy ears” (line 27) facilitates the overgrowth of yeast. These admissions are then linked sequentially and logically with “and” to the veterinarian’s straightforward assertion, “this is yeast” (line 30). This diagnosis is supported empirically by the veterinarian sniffing the patient’s ear and presumably identifying the telltale odor as a clinically relevant sign.

The confident design and delivery of this announcement upholds the epistemic authority of the veterinarian to diagnose. The announcement contrasts with the uncertain framing of the previously contested hypothesis that a food allergy may be implicated in the recurrent nature of the infection. In the absence of client endorsement of an attempt to take a nutritional approach to the prevention of future ear infections, the veterinarian focuses exclusively on treating the current one. On the basis of the unchallenged diagnosis, after this segment ends, the veterinarian recommends combination therapy, consisting of anti-inflammatory and anti-fungal medications, which the clients accept.

In Case 4, the client eventually accepted the veterinarian’s proposal to immediately switch the patient from puppy to adult food. Extract 4.2 opens with the client’s justification for having suggested that they finish the current bag of puppy food before effecting the change (line 16).



**Extract 4.2.**


16

C: **Cuz it says up to two years on the bag**

17

V: Yeah I know but

18

   (0.4)

19

V: Usually with-especially with a large dog?

20

   (0.4)

21

V: after they’re about twelve months old?

22

C: Oh [okay.

23

V:    [they get them over to adult so

24

C: [Okay.

25

V: [Not a big deal?

26

   I mean it’s not like she’s heavy or any[thing?

27

C:                                          [Noo

28

V: An’ ob-obviously she’s good and active?




The veterinarian delicately disagrees with the client’s reasoning. The acknowledgment of the credibility of the client’s report about feeding instructions on the dog food package (“Yeah I know,” line 17) is immediately appended with the disjunctive “but”; following a 0.4 s gap (line 18), the veterinarian introduces contrasting medical norms associated with the particular health needs of large breed dogs (line 19). The *self-repair* [85] whereby “especially” replaces “usually” serves to emphasize the distinctiveness of the health risk for this category of dog and, concomitantly, this patient.

The veterinarian then mentions the recommended age cut-off for large breeds “after they’re about twelve months old” (line 21) at which point the client acknowledges this information with “Oh okay” (line 22). The “oh” preface indexes the shift in the client’s epistemic state; once ignorant of the special nutritional requirements for large-breed dogs like the patient, the client is now informed. The veterinarian uses the generic third-person pronoun “they” (line 23) to describe what the body of experts in veterinary care do in terms of normative practices of switching large-breed dogs to adult food (line 23). Thus, the veterinarian counters client resistance and upholds the recommendation via the consensus of veterinary medical experts. The client acknowledges this generalized recommendation (line 24) in overlap with the veterinarian who uses reassuring assessments of the situation (“not a big deal,” line 25) and of the patient (“not…heavy or anything,” line 26) to position this recommendation as preventative in nature and to defuse any possible inference that the patient is already showing signs of overweight associated with excess nutrition from having eaten puppy food until now. The client promptly agrees with this last assessment (line 27). The veterinarian’s comment that the patient is “good and active” (line 28) further manages the face threat of the recommendation with a positive assessment that, by implication, constructs the client’s care of their pet as beyond reproach; this comment also foreshadows the veterinarian’s subsequent description of adult foods formulated for active dogs. That comes after the end of the extract and elicits a request from the client for recommended brands.

As with Case 4, Case 5 led to client acceptance of the veterinarian’s proposal to change the main food. Extract 5.2 opens with the client describing the preference exhibited by Rascal, another cat in the household, for his current food (lines 34, 35).



**Extract 5.2.**



34


C: Yeah cuz Rascal’s been eatin’ hard kibble=Yeah **he**

35

   **likes the stuff he has**.

36

V: Good.

37

C: So.

38

V: Okay [so

39

C:      [(Now even) a free bag (would be nice) just to

40

   see if he likes it=cuz I I’ll see by the price and I’ll

41

   (portion) [it out (and see how it is.)

42

V:           [Yeah and it’s not bad too because if you

43

   think um with the the prescription diets they are

44

   going to be a bit more expensive=

45

C: =Mm hm. *((C nods))*

47

V: than a lot of the grocery foods but. you don’t usually

48

   have to feed as much of them just cuz they’re a bit

49

   higher quality so more nutrient dense?

50

C: Hm= *((C nods))*

51

V: =So you don’t have to feed as many kib[bles?

52

C:                                        [Or you could

53

   mix them with a low [price brand.

54

V:                     [Yeah or you could mix them

55

C: Okay

56

V: And as well if you factor in that you’re using the 

57

   dental diet hopefully trying to extend the time

58

   between a dental cleaning then that definitely saves

59

   money that way.=If you’re putting (that) a bit more. So

60

C: Okay.

61

V: Definitely I’ll give you the price so you can think

62

   about it.

63

C: Yeah ((V puts on stethoscope))

64

V: And if we can just get him over here




The veterinarian accepts this state of affairs; the positive assessment “Good” (line 36) deftly avoids taking up the client’s concern about Rascal’s liking of his current food as a problem. The client’s stand-alone “so” prompts further action by the veterinarian who launches a turn (line 38) and promptly drops out before turn completion when overlapped by the client who performs a marked acceptance (lines 39–41) of the previous offer of the free food sample, using a positive assessment (line 39) and incorporating as contingencies the two previously mentioned possible obstacles to adherence: the food preference of the absent pet and the cost of the proposed food (line 40).

The veterinarian then shares information about the dental diet that is designed to reduce the client’s concerns about the issue of cost while simultaneously demonstrating the veterinarian’s sensitivity to this as a legitimate issue (lines 42–44; 47–49). The construction of the cost-related scenario of feeding the dental diet is evaluated using a mitigated negative assessment “not bad” (line 42); the cost of the prescription food relative to grocery store foods is downgraded to “a bit more expensive” (line 44), which is acknowledged by the client (line 45). In reassuring the client that cost savings are likely given that smaller portions can be served (lines 47–49), the veterinarian appears to build on the client’s previously mentioned intention to portion out the free sample in an exploratory fashion (lines 40–41).

On lines 52 and 53, the client issues a candidate option of another way of rationing the dental diet to manage barriers to adherence: mixing the dental kibbles with the currently used main food. This proposal is designed as a turn increment; it begins with “or” which constructs this turn as an add-on alternative to the veterinarian’s previous turn, the negatively worded utterance on line 51 regarding necessary portion sizes. This proposal by the client represents a further instance of marked acceptance of the suggested dietary change. High engagement in the diet discussion is demonstrated by the client’s own independent problem-solving regarding the obstacles to adherence. The epistemic authority of the veterinarian as the medical expert to determine the acceptability of the proposed nutrition-related practices is upheld by the veterinarian’s ratification of the client’s proposal (“Yeah,” line 54); moreover, the veterinarian’s repetition of the proposal as if it is their own (line 54), which the client accepts (line 55), demonstrates strong affiliation with the client.

The veterinarian offers further information about the cost-saving benefits of mixing the current food with the dental diet: the postponement of expensive dental cleaning (lines 57–59), which the client acknowledges with “Okay” (line 60). The veterinarian then reiterates the promise to provide the client with the price of the dental food; the clause “so you can think about it” (lines 61–62) explicitly frames the proposal to use the dental diet not as an agreed-upon treatment plan but rather as something merely under consideration by the client. After the segment ends, near the end of the appointment, the veterinarian promises to obtain the sample bag of food, to which the client responds with “Okay.”

In sum, conversation analysis of veterinarians’ uptake of clients’ active resistance demonstrated varied orientations to the barriers to adherence raised by clients in reaction to the original dietary proposals. Veterinarians responded to the superior epistemic access of clients when their resistance involved the disclosure of new information about patients’ or clients’ past or current experiences and preferences. Subsequent proposals were typically modified in these cases so as to incorporate the concerns of clients while still maintaining a focus on patient health. Veterinarians would suggest other options, including non-nutritional ones, for addressing or preventing the health concerns motivating the original proposal for dietary change. In light of the uncertainty regarding patient adherence and other obstacles, veterinarians’ subsequent proposals were sometimes downgraded to suggestions to merely consider nutritional modification or were designed as options in a way that retrospectively constructed the initial proposal as one choice among several, rather than as the only recommended course of action. These constructions mitigated the deontic authority of veterinarians to expect clients and patients to follow the proposed strategies.

When clients’ resistance manifested as questions or comments that revealed a lack of medical knowledge about the relationship between the dietary proposal and patient health, veterinarians upheld their own epistemic authority by educating clients, explaining diagnoses where appropriate, and providing reasons for the dietary proposal and/or alternative strategies, sometimes including evaluations of the relative effectiveness of the various approaches. Veterinarians did not explicitly inquire about clients’ interest in addressing the patient’s health issue or in a diet-related remedy. Clients deferred to the superior epistemic status of veterinarians by accepting their diagnoses, clinical interpretations, and assessments of possible treatment options. While the previously presented Case 3 might appear to be an exception, it is important to note that the veterinarian in that case epistemically downgraded the causal link between the patient’s health issue and the current diet to a mere possibility when suggesting a dietary change; in contrast, the diagnosis of a yeast infection in the patient, treatable with prescription medications, was presented as a clinical certainty.

## 4. Discussion

Conversation analysis has shown that institutional talk like veterinary dietary discussions is built on the bedrock of ordinary conversation; this is because people in professional settings draw on the same interactional resources used for everyday communication [86]. As we have seen in the present study, those resources may help minimize disagreements and facilitate the smooth progression of the conversation, but they are not always optimal for the specialized activities and goals of veterinary appointments. The present research on clients’ active resistance to long-term dietary change is one of a series of investigations of real-life veterinary–client interactions [51,52,87] aimed at informing practice guidelines for the nutritional assessment and weight management of dogs and cats [3] and, hopefully, clinical communication training in veterinary education. The implications of the present study for veterinary clinical practice become clear when the results are situated in the context of extant research and the best-practices literature on veterinary communication in the area of pet nutrition.

### 4.1. The Prevalence of Active Client Resistance

In our corpus of data, there were slightly more cases of initial active client resistance (*n* = 23) to veterinarians’ proposals for long-term nutritional modification than cases of acceptance and passive resistance (*n* = 19). This is not necessarily surprising, given the findings of the landmark study previously cited [13]. An article promoting effective strategies for talking with clients about dog and cat nutrition normalized the potential for client resistance to dietary recommendations by suggesting that such resistance could very well arise at several decision points in every pet’s lifespan: what puppy or kitten food to use, when to switch a pet to adult food and senior food, and what foods to select at those transition times [88]. Given the importance of sound nutrition for animal health, of concern is a recent survey in which 41.5% of veterinary clinicians reported that client resistance to changing their brand of pet food deterred clinicians from initiating nutrition discussions in wellness appointments [89]. That finding seems bound up with the intrinsically face-threatening nature of the topic: “Few subjects are as emotionally loaded and full of personal opinions as what and how people feed their pets” ([90], p. 907). Adding to the complexity is other survey research indicating that clients view their veterinary healthcare team as the most crucial source of information when making decisions about changing their pets’ diet [91] and purchasing pet food [92].

But client resistance to dietary recommendations need not be a foregone conclusion. A conversation-analytic study in human medicine found that primary care physicians’ recommendations for new drug treatments produced fewer instances of patient active resistance in cases in which physicians first requested information about prior medication use than in cases in which such preliminary inquiries did not occur [93]. Patient information gathered before delivering the recommendations permitted physicians to tailor their communications to the particular patient and avoid resistance on the following bases: patients currently taking the recommended drug; patients having previously tried the drug and found it to be ineffective; patients’ aversion to taking the drug; patients’ concerns about harmful drug effects; and affordability [93]. Such findings resonate with many of the barriers to adherence disclosed by clients in the present study. Thus, the prevalence of active client resistance in our study might have been reduced had veterinarians engaged in preliminary information-sharing and information-gathering with clients before proposing dietary changes.

### 4.2. The Importance of Nutritional History-Taking

Nutritional assessments, including thorough nutritional histories [3,52], should be conducted with clients so that veterinarians have the necessary information to customize their treatment recommendations to the particular client and patient, and reduce the likelihood of resistance. In certain cases in our study, veterinarians were unaware of the patient’s current diet, such that they recommended foods that clients were already feeding their pets. In other cases, veterinarians did not know about competing health concerns in the patient, a history of gastrointestinal difficulties associated with dietary changes, or if the patient had previously rejected the specific food that was recommended. In this regard, the finding that there were proportionally more cats than dogs in the active resistance collection was partly due to species-specific difficulties switching cats to a different dry food or introducing canned food: feline neophobia [94]. In other cases, veterinarians lacked important information about feeding management in relation to the patient’s environment [3] (e.g., differential food preferences among multiple pets and/or risks to the health of the patient or other pets in the household due to food competition and shared feeding scenarios). Such information can be helpful to veterinarians in developing individualized feeding plans for patients that could overcome potential obstacles to adherence [3].

Of note in our study is that veterinarians typically accommodated clients when their resistance was based on particular past or present experiences of the patient, such as dispreference for the recommended food, potentially conflicting health concerns, and possible side effects. This finding is similar to one reported in a conversation-analytic study about parents’ resistance to clinicians’ treatment recommendations for their children diagnosed with epilepsy in which clinicians accommodated parents (by backing down, giving concessions, and providing options) when parental resistance was based on the past experiences of their child, rather than on an anti-medication position [42].

### 4.3. The Importance of Educating Clients

Veterinarians need to educate clients about patient health issues before making recommendations to address them [52]. In support of this, clients surveyed at wellness appointments for one study indicated that veterinarians’ statements about their pet’s health would be the type of communication that would most influence them to change their pet’s diet [91]. Some clients in the present study made comments or asked questions suggesting that the dietary proposals were irrelevant because they either believed that current food targeted by the proposals did not have a deleterious effect on pet well-being or they were afraid that the new food intended to support pet health would inadvertently cause other health problems. Resistance thus arose because clients lacked knowledge about nutrient-sensitive or diet-induced health disorders that could be prevented or ameliorated with nutritional modification [2,3]; other clients implied that the recommended changes were unnecessary because they were using equivalent food items or alternative practices to address the health concern, approaches that veterinarians subsequently deemed to be problematic or less effective forms of treatment than the proposed change.

In accord with their epistemic superiority regarding expert knowledge about animal health, as compared with other grounds for resistance, veterinarians were less likely in these cases to abandon or revise their initial dietary proposals; clients were more likely to ultimately accept them when their resistance was tied to a lack of medical knowledge about the benefits of the food change. Following resistance, veterinarians typically justified the prior proposal by informing the client about the health concern and/or about why the proposed dietary modification was the most effective strategy for addressing the concern. This dovetails with results of a systematic review [95] of conversation-analytic studies of primary care doctor–patient talk about health behaviour in which the physician’s linking of a proposed behaviour change to a health issue that was salient for the patient was an effective tool for managing client resistance [95].

### 4.4. The Importance of Providing Multiple Treatment Options

When it is appropriate to do so, veterinarians should situate dietary recommendations within a list of treatment options and provide information about their benefits and drawbacks, including their relative effectiveness [3,52]. In the present study, veterinarians often accommodated clients by proposing alternative strategies in the aftermath of resistance to the dietary proposals. That the provision of multiple treatment options is important in addressing patient health is highlighted by our finding that in over 40% of the active resistance cases clients wound up accepting an alternative strategy, sometimes non-dietary, to support pet wellbeing. However, these strategies tended to be proposed in the wake of client resistance to the dietary proposals, often delivered in a series of single proposals for clients to consider individually, one by one in sequence, rather than as a range of choices to be evaluated as a set. In this sense, in the present study, there was more option-*chaining* (with client resistance to one option occasioning the mention of another one) than comprehensive option-listing. Clients most often accepted a single alternative strategy with “Okay,” rather than articulating a choice among strategies. As previously noted, the sequential presentation of proposals by some veterinarians camouflaged client disagreement by recasting the previously rejected dietary proposal, not as a singular recommendation, but as one of a series of possibilities.

Of relevance is conversation-analytic research in human medicine about neurologists’ recommending and option-listing approaches to treatment discussions with outpatients [95]. Results indicated that option-listing oriented strongly to patients’ right to choose and also structurally expected patients to explicitly select from the list of options [96].

### 4.5. The Importance of Soliciting Clients’ Perspectives

In order to reduce the likelihood of client resistance and to ensure that treatment recommendations are tailored to the individual client and patient, veterinarians should solicit clients’ perspectives, including whether they are open to discussing their pets’ nutrition [3,52]. This is critical in a medical context in which veterinary clients, as compared with patients in human medicine, have many more options available to them, including the decision to eschew treatment [48]. Information can be gathered about the client’s interest in addressing their pet’s health problem, their perceptions about the relevant health issue, their concerns about and commitment to nutritional change and, if appropriate, their interest in hearing about a specific, recommended food [3,52]. This solicitation begins with nutritional history-taking and proceeds through the various stages of dietary discussion in an iterative fashion [3,52].

It is noteworthy that, in our corpus, veterinarians did not explicitly invite clients’ perspectives. Either these were presumed to align with those of the veterinarian as implied by clients’ acceptance of veterinarians’ dietary proposals, were withheld in instances of passive resistance, or “leaked out” as active resistance in the aftermath of veterinarians’ dietary proposals. In line with the conversation-analytic principle of minimizing disagreement, soliciting a client’s perspective may seem risky in potentially inviting their disaffiliation with the veterinarian’s medical opinion, should differences in perspective be exposed. However, also in accord with the principle of minimizing disagreement, some discussions in our collection were seemingly protracted because veterinarians kept obliquely pursuing client engagement (that was not forthcoming), providing multiple pieces of nutrition-related information instead of inviting clients directly to share questions, ideas, and concerns. Asking about possible sources of resistance normalizes their existence. Moreover, and unlike patients and parents in studies in human medicine involving prescription drug recommendations [33,34,35,36,37,38,39,40,42,93], veterinary clients exercise considerable deontic authority in being able to access a plethora of over-the-counter food choices for their pets outside of clinic-only diets.

The issue of potential patient noncompliance with a dietary switch, and the stresses associated with the change for the client, loom large in the veterinary context. Conversation-analytic studies have shown how veterinarians and clients manage interactions with animals as non-cooperative patients [49,50]. In this regard, the results of our previous study [52] and the present one that investigated veterinarians’ responses to client resistance may more closely resemble conversation-analytic work on physicians’ advice to patients about lifestyle changes [97] rather than physicians’ drug recommendations. In the latter scenario, doctors alone have the prerogative to prescribe [42] and patients and patients’ parents sometimes pursue prescriptions even when contraindicated [34,35,36,38,42]. In one study on lifestyle modifications, physicians advising patients about changing their behaviour avoided the use of directives, employing conditional “if” statements, information-sharing, and option-sharing to uphold the agency and autonomy of the patient in deciding to change their behaviour [97]. In a different study, physicians similarly employed conditionals when offering support to patients on smoking cessation, the design of which oriented to doctors’ limited agency in shaping patient behaviour [98]; patients’ perspectives on, and future intentions about, smoking cessation following such offers were frequently unclear [98].

In the present study, in spite of—and in other cases because of—veterinarians having gained important information from clients through their displays of active resistance, the design of the final proposals often oriented to possible nonadherence. Veterinarians acknowledged many contingencies, including client and patient preferences, that made the adoption of dietary modification or alternative strategies uncertain (except when the alternative strategy involved prescribed medication). Such formats included epistemically downgraded proposals to consider dietary change in the future, information-sharing about options, and generic advice (e.g., to merely keep patients’ teeth clean or to keep the patient from gaining weight). Research in veterinary and human medical communication suggests that unclear, vague recommendations can reduce adherence [30,95]. Recent practice guidelines contain step-by-step communication practices that can shape clear nutritional recommendations specific to the particular client and patient [3].

Veterinarians’ accommodation of relevant potential obstacles to adherence and the mitigated design of the final proposals were likely critical to the outcomes in our study whereby 78% of clients either ended up accepting the veterinarian’s original dietary proposals or an alternative strategy the veterinarian proposed to address or prevent patient health problems. Clients usually responded to these proposals with a final-intonation “Okay” and no further elaboration. This is in line with conversation-analytic research demonstrating that patient acceptance of typically designed medical recommendations takes the form of a preferred turn shape, usually “Okay” [96]. While such a response to a recommendation furthers the smooth unfolding of the appointment, this sort of adjacency pair does not facilitate active patient participation in medical decision-making the way that option-listing does, whereby patients are invited to choose between treatment strategies. In contrast, “recommending turns do not *invite* patients to do more than simply accept a decision” ([96], p. 881). Interestingly, the four cases in our study in which clients demonstrated a more elaborate acceptance of veterinarians’ recycled dietary proposals following active resistance involved veterinarians inviting clients to take free samples of the recommended food or to choose between two different treatment options in the wake of resistance. This finding shows that, despite some of the interactional challenges posed by initial active resistance, it does not necessarily forecast ultimate client disaffiliation with nutritional modification. Active resistance cases often included more client engagement, including client counterproposals accepted by veterinarians, than were involved in non-resistance cases.

### 4.6. A Collaborative Approach to Dietary Decision-Making

As described in our companion study [52] and as suggested above, a veterinarian can create the foundations for collaboration with the client in treatment decision-making by taking the necessary time to engage the client actively, and cyclically, as a partner during the dietary discussion (see also [3]). Information-sharing by both the client and the veterinarian, who have their own domains of expertise, is crucial. As we have mentioned, thorough history-taking with the client is essential so that the veterinarian avoids suggesting treatment strategies that are ill-suited to the patient and/or the client or other pets in the home. The veterinarian then needs to educate the client about the patient’s health problem and ask the client explicitly about their level of interest in ameliorating or preventing the problem [52]. A range of treatment options should be described, with the veterinarian seeking the client’s perspective on the options and inviting their questions and concerns [52]. In this way, potential client resistance is surfaced early and as a topic of discussion valued by the veterinarian rather than as an impediment. Should the veterinarian’s treatment recommendation involve the introduction of a new food, at this juncture, the veterinarian can make a clear recommendation at a level of generality higher than a particular product name or brand [52] (e.g., a dental diet or a vegetarian diet). The veterinarian should again invite the client’s perspective on the recommendation, including any concerns they may have [52]. If the client seems receptive, the veterinarian can then provide various product options, discussing the pros and cons of each, and again solicit the client’s perspective on the product choices [52]. Depending on the client’s response, the veterinarian can recommend a particular product, making sure to check in again with the client and to jointly create an action plan [52]; this would include steps for introducing the new food and for following up with the client to assess the success of the plan and possible need for modification [52].

### 4.7. Strengths and Limitations

One strength of the present study is that it provides evidence-based findings from the analysis of real-life interactions between veterinarians and clients to inform clinical practice and communication training in veterinary education regarding the nutritional assessment of dogs and cats and effective treatment recommendations. Another strength is that we examined not only the design, sequential organization, and grounds of clients’ demonstrations of active resistance to veterinarians’ initial long-term dietary proposals; we also studied veterinarians’ uptake of client resistance and the final outcomes of nutritional decision-making so that the consequentiality of client resistance and the implications for adherence to nutritional recommendations could be better understood.

Nevertheless, certain limitations regarding our research data require mention. Unfortunately, the study did not include independent follow-up measures of adherence with agreed-upon treatment strategies. Other limitations are associated with our sample. Successful conversation analysis is frequently based on small samples, in part due to the labour-intensiveness of the methodology. However, it would be beneficial to expand the current data set by collecting additional cases from different clinics with which to compare the interactional regularities and variations identified in our study. Samples from multiple jurisdictions would also help, given the small geographic area of eastern Ontario, Canada, from which our sample was taken. Conversation analysis of large samples from England and the United States of patient resistance to treatment recommendations in primary healthcare encounters demonstrated key cross-national differences in the resistant stances that patients took toward the recommendations [41].

The time frame of 2006 in which our data were collected is another possible limitation. Widespread changes since then include the following: the rise of online purchasing in the pet food industry, particularly since the inception of the recent pandemic [99]; the ever-expanding number of choices available in commercial pet foods [99]; premiumization and the increasing focus of pet owners on the healthiness of their pets’ diets [99]; the associated humanization of pet food products, reflecting social trends in human dietary changes [99,100]; alternative diets [101], including organic and natural products [99,101]; grain-free options [100]; home-prepared diets [3]; plant-based diets [88]; and raw food diets [3,88]. There is also emerging medical information about diet-associated health risks (e.g., canine dilated cardiomyopathy, [101]) and guidelines for weight management [102]. With respect to feeding management, technological advances mean that clients can buy increasingly sophisticated automated feeders employing microchips or collars to restrict access of a food to one pet in multi-pet households [103].

Since the time our data were collected, these developments may well be reflected in shifts in the content of some patients’ diets, the kinds of preferences clients express for particular foods, the nature of the expert information veterinarians share with clients to promote dietary change (including the risks and benefits of current pet food trends), and the increased availability of non-nutritional strategies to overcome obstacles to dietary modification. Nevertheless, the results of recent conversation-analytic studies [42,92] in human medicine on parents’ and patients’ resistance to doctors’ treatment recommendations indicate that many of their findings, including grounds given for resisting recommendations, resonate with the results of our own study. This suggests the stability and ubiquity of the phenomenon of client resistance to dietary recommendations identified in the present study and the ongoing relevance and applicability of its findings.

## 5. Conclusions

The powerful impact of nutrition on animal health requires effective diet-related communication in veterinary medicine. Published work documenting veterinary clients’ nonadherence to nutritional recommendations and its likely outcomes has relied on the use of client and clinician surveys (e.g., [13,89,91]) such that little has been known about how client resistance to veterinarians’ dietary treatment recommendations is constructed in the language of actual appointments or how clinicians typically respond to nonadherence. The present study attempted to overcome these limitations using the qualitative research methodology of conversation analysis. Because it focuses on patterns of turn-taking and the wording of individual turns, conversation analysis can pinpoint trouble spots in interactions between veterinarians and clients, the significant features of the preceding conversational actions that shaped them, and the interactional consequences for how these trouble spots were subsequently managed. It is hoped that the results of this study demonstrate the value of this method in supporting effective veterinary communication training and professional practice guidelines, including those related to nutritional assessment and shared decision-making.

## Figures and Tables

**Table 1:** Characteristics of appointments (patient species and type of appointment) based on presence or absence of active resistance in clients’ responses to veterinarians’ proposals for long-term dietary change.

	No. (%) of Appointments with and without Active Resistance		
Characteristics	Active Resistance *n* = 21 (60%)	No Active Resistance *n* = 17 (49%)	Total *n* = 35
Patient Species			
Cat	13 (62)	6 (35)	17 (49)
Dog	8 (38)	11 (65)	18 (51)
Appointment Type			
Wellness	14 (66.7)	14 (82.3)	25 (71)
Problem	6 (28.6)	3 (17.7)	9 (26)
Follow-Up	1 (4.8)	0 (0)	1 (3)

**Table 2:** Grounds on which clients actively resisted veterinarians’ proposals for long-term dietary change, including prevalence and examples of client responses.

Grounds for Resistance	No. (%)	Example	Dietary Change Proposed in Example
A. Patient food preference or dispreference re. current or proposed diet (CHANGE NOT FEASIBLE)	7 (22.58)	*1. What do you use? What are you suggesting then, something* *they’ll eat?* *2. Cuz she’s asking all the time for cookies.*	Introduce dental food Replace high fat treats with baby carrots
B. Multi-pet feeding issues (food preference or dispreference of other pets, food competition, negative health consequences for other pets) (CHANGE NOT FEASIBLE)	6 (19.35)	*3. That’s why yeah it’s hard when for when there’s three.* *4. Well cuz she seems hungry and asks for food.*	Reduce amount of dry food, increase in canned for only certain pet in multi-pet home Switch to light formulation for only certain pets in multi-pet home
C. Current enactment of other strategies to address health concern (CHANGE NOT NECESSARY)	5 (16.13)	*5. We try ta give em like tartar control treats and stuff like that as well.*	Introduce dental food
D. Current enactment of proposed dietary change (PROPOSAL NOT NECESSARY)	3 (9.68)	*6. Yeah I know. Actually I have her on that so.*	Switch to senior formulation
E. Concern about inducing new medical problem or worsening current comorbid condition (CHANGE INAPPROPRIATE)	2 (6.45)	*7. Oh yer kidding. I thought canned food was bad.*	Introduce canned food
F. Questioning of etiological role of current food in patient medical condition (CHANGE INAPPROPRIATE)	2 (6.45)	*8. I don’t think that sticks to the teeth as much, do you think?*	Eliminate canned food
G. Cost of proposed food (CHANGE NOT FEASIBLE)	2 (6.45)	*9. How about a price comparison between the {Current Brand Name} and yours?*	Switch to higher-quality diet
H. Side effects of food change in patient that would be aversive for client (CHANGE NOT FEASIBLE)	1 (3.23)	*10. When I change anything her gas is awful.*	Introduce dental food
I. Client preference for or emotional attachment to current food (CHANGE NOT FEASIBLE)	1 (3.23)	*11. So if I wanna give out cookies what I’m gonna do?*	Replace high fat treats with baby carrots
J. Client lack of familiarity with proposed food (CHANGE NOT FEASIBLE)	1 (3.23)	*12. I’m not sure what the ones that yer talking about look like.*	Introduce dental food
K. Feeding information on current food packaging in conflict with proposed food change (CHANGE INAPPROPRIATE)	1 (3.23)	*13. Okay. We’ll finish this bag and she’ll go on adult food.*	Switch from puppy to adult formulation
Total	31 (100%)		

**Table 3:** Final outcomes of decision-making trajectories in cases with client active resistance to veterinarians’ proposals for long-term dietary change.

Outcome	No. of Cases (%)
Acceptance of Alternative Strategy (Dietary/Non-Dietary)	10 (43.5)
Acceptance of Initial Dietary Proposal	8 (34.8)
Ratification of Modified Client Counterproposal	2 (8.7)
Postponement of Decision	2 (8.7)
Outcome Unclear	1 (4.3)
**Total**	23 (100%)

## Data Availability

Because informed consent for public posting of their videotaped data was not provided by the research participants at the time of data collection, and to protect their privacy as required by approved ethics procedures, public access to the data is not available. Excerpts of anonymized written transcripts are included in this publication as part of the standard validation practices in conversation-analytic research.
